# Extracellular ionic fluxes suggest the basis for cellular life at the 1/*f* ridge of extended criticality

**DOI:** 10.1007/s00249-020-01430-3

**Published:** 2020-03-24

**Authors:** Mariusz Pietruszka, Monika Olszewska

**Affiliations:** Faculty of Natural Sciences, Institute of Biology, Biotechnology and Environmental Protection, University of Silesia in Katowice, 28 Jagiellonska Str., 40032 Katowice, Poland

**Keywords:** Extracellular ion fluxes, Optimum growth, Pollen tubes, Resonance curve, Self-organised criticality, Scale-free dynamics

## Abstract

**Abstract:**

The criticality hypothesis states that a system may be poised in a critical state at the boundary between different types of dynamics. Previous studies have suggested that criticality has been evolutionarily selected, and examples have been found in cortical cell cultures and in the human nervous system. However, no one has yet reported a single- or multi-cell ensemble that was investigated ex vivo and found to be in the critical state. Here, the precise 1/*f* noise was found for pollen tube cells of optimum growth and for the physiological (“healthy”) state of blood cells. We show that the multi-scale processes that arise from the so-called critical phenomena can be a fundamental property of a living cell. Our results reveal that cell life is conducted at the border between order and disorder, and that the dynamics themselves drive a system towards a critical state. Moreover, a temperature-driven re-entrant state transition, manifest in the form of a Lorentz resonance, was found in the fluctuation amplitude of the extracellular ionic fluxes for the ensemble of elongating pollen tubes of *Nicotiana tabacum* L. or *Hyacintus orientalis* L. Since this system is fine-tuned for rapid expansion to reach the ovule at a critical temperature which results in fertilisation, the core nature of criticality (long-range coherence) offers an explanation for its potential in cell growth. We suggest that the autonomous organisation of expansive growth is accomplished by self-organised criticality, which is an orchestrated instability that occurs in an evolving cell.

**Graphic abstract:**

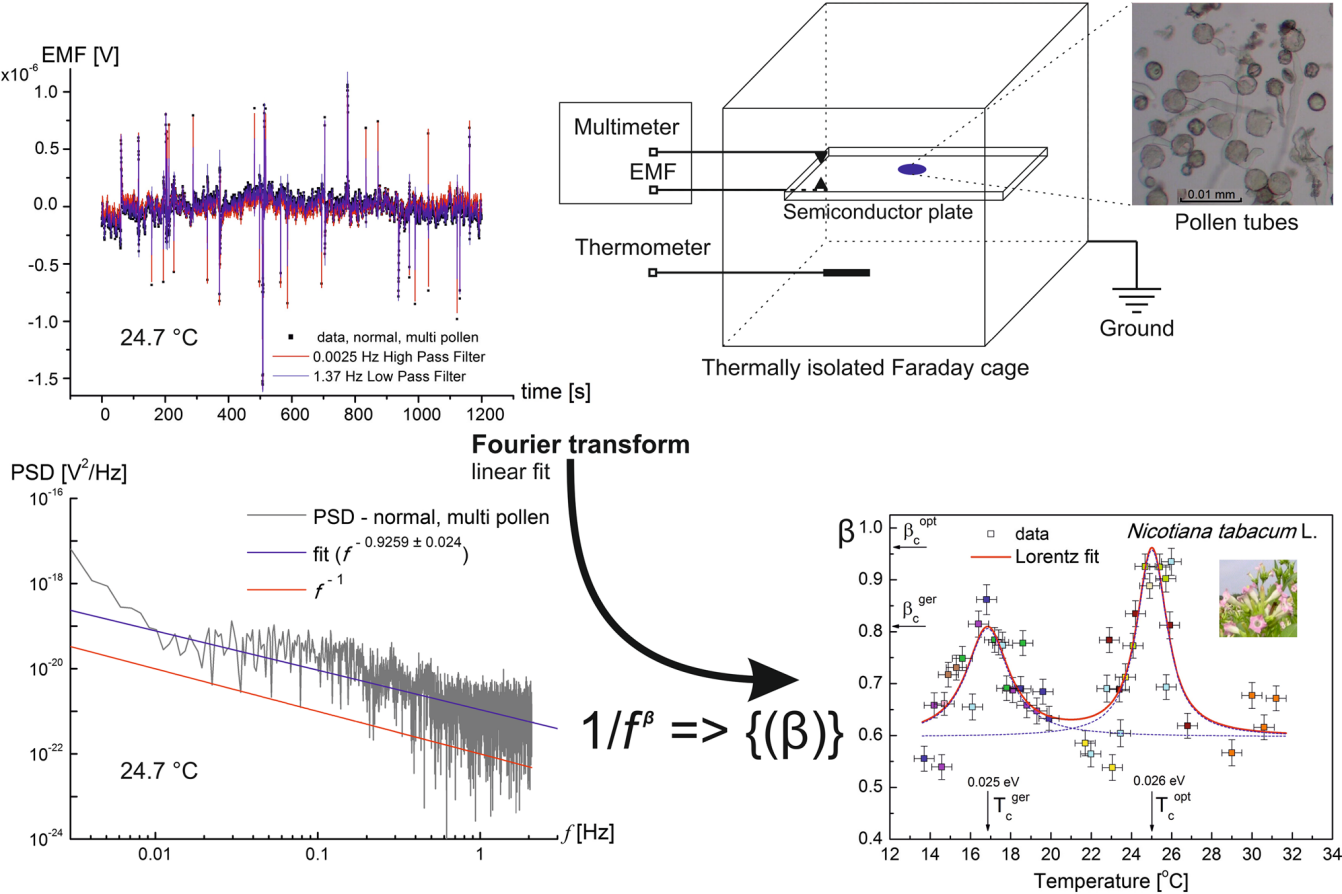

**Electronic supplementary material:**

The online version of this article (10.1007/s00249-020-01430-3) contains supplementary material, which is available to authorized users.

## Introduction

The second law of thermodynamics requires that the overall entropy change be nonnegative for any real process. Non-equilibrium thermodynamics is helpful in describing the processes of highly ordered non-equilibrium structures, because it provides a way to rationalise the local decreases in entropy that are necessary for the formation of extraordinarily ordered entities such as living organisms. The entropy of an adiabatically isolated system never decreases. On the other hand, a living organism is not an isolated system. It gets nutrients from the exterior; that is, there is an exchange of energy or matter (information) with the environment. Hence, we have to consider an investigated system (here, a plant cell) together with its environment, which restores the balance by an increase of entropy or by an increase of disorder (Haynie 2008; Himeoka and Kaneko 2014; Barbacci et al. 2015 for review). An object that conforms to this view is a living cell of a plant, here a pollen tube.

A pollen tube is a tubular structure that is produced by the male gametophyte of flowering plants when they germinate. In flowering plants, the pollen tube acts as a conduit to transport the male gamete cells from the pollen grain in the stigma to the ovules at the base of the pistil. Pollen tubes are used as a model for understanding plant cell behaviour. In this work, a pollen tube is an experimental system that is allowed to grow in cell length in an appropriate bath medium which provides the necessary nutrients. In what follows, we want to study whether optimal growth coincides with power-law voltage fluctuations of ion channels generating extracellular ionic fluxes that can be measured (Pietruszka et al. 2018). Physiological time series (voltage) may exhibit self-affine (self-similar) properties (Hardstone et al. 2012 and papers cited therein). In self-affine processes, the statistical distribution of the measured quantity follows a power-law function, which is the mathematical function without a characteristic scale and is, therefore, called “scale-free”. Scale-free phenomena are described by the exponent of a power-law function, because it captures the relationship between fluctuations on different scales. The power-law function makes a straight line in double-logarithmic coordinates; the slope of this line is the exponent of the power law, here: *β*.

This ‘system’ (a pollen tube) can exchange its ion mass and charge (energy) with ‘the rest of the world’ (here: an incubation medium) and store or lose information as it grows (eventually growth ceases altogether and entropy increases). From our current and recent investigations (Fig. 4 in Olszewska et al. 2018), it appears likely that this non-equilibrium process is most efficient in the ‘normal’, i.e., evolutionarily selected mode of growth. Note that the pH-growth rate cross-correlations are strongest under the control conditions (ibid.), which correspond to ‘normal’ growth.

Criticality occurs when a system is poised at the point of a dynamic instability (see Cocchi et al. 2017 for review). The present study shows that apparently chaotic ion-channel noise follows the 1/*f* ordering rule for the full range (about three decades) of the relevant frequencies *f* in this case*.* The latter statement probably means that normal (regular) cell life exists on a narrow ridge with a spectral density of 1/*f*^*β*^ where the spectral signature *β* = 1. Yet, a similar feature concerning the power spectral density (PSD ~ 1/*f*^*β*^) has already been recognised in theory (Bak et al. 1988) as self-organised criticality (SOC). Even though power laws (see Newman, 2005 for review) do not imply self-organised criticality, the symptoms of criticality, or even SOC behaviour have been identified in the human brain and many other systems (Dutta and Horn 1981; Hesse and Gross 2014; Markovic and Gros 2014). Moreover, in a model of neuronal activity, an association was observed between self-organised critical dynamics and several neurobiologically realistic features of structural connectivity (Rubinov et al. 2011). Self-organised criticality is based on the idea that complex behaviour can develop spontaneously in certain many-body systems whose dynamics vary abruptly (Jensen 1998). In general, it seems that the biological rhythm is basically subject to 1/*f*^*β*^ fluctuations from the cellular to behavioural levels; no (external_MP_) fine tuning is necessary to generate 1/*f* noise in nature (Bak et al. 1988). However, no actual measurements on a single (or multiple) living cell(s) have been performed with a specific outcome of *β* = *β*_c_ = 1, which henceforth can possibly be treated as the signature of the evolutionary process or optimal conditions in which a particular life form was selected to operate in. It is quite intriguing that the self-organisation of living systems towards criticality leads to a pure 1/*f* spectrum with exponent *β* = l (see the comment after Eq. 6 in Tang and Bak 1988), which corresponds to simple diffusion for the normal mode of growth.

Beyond this essential background context, a practical one arises. A deviation in parameter *β* from the *β* = 1 rule (power spectral density as being inversely proportional to the frequency in the log–log scale) can probably be used to detect any abnormal development of a cell (Lancaster et al. 2016). This, in turn, can possibly be used in human health care for labelling disease entities by the value of their *β* exponent—deviations from *β*_c_ = 1 criticality could be symptomatic or causative for certain pathologies (Hesse and Gross 2014). Normal development or disease can lead to differences in the scale-free amplitude modulation of oscillations (Hardstone et al. 2012). A potential role in testing drugs or in facilitating the identification of altered metabolic states is also foreseen as being an inevitable consequence of the *β* = 1 exponent. Summarising, *β* = 1 can be treated as a possible indicator of health.

The term “criticality” has been adopted to denote both noise-induced phase transitions and SOC with no clear connection with the traditional concept of phase transition, that is the transformation of a thermodynamic system from one state of matter to another (Lovecchio et al. 2012). However, self-tuned phase transitions can (and do) exist in nature—the core idea of SOC was clearly enunciated in Bak and Chen (1989) and was presented as the dynamic origin of spatio-temporal fractals in nature. If this is the case, at a microscopic scale, the ionic “avalanches” that are controlled by the level of the chemical potential (the “hour-glass” or metronome) may produce a macroscopic outcome—the temporal (Pietruszka 2019) or spatial (Hemelryck et al. 2018) oscillations of pollens. However, we must admit that the data that we currently gathered (Pietruszka et al. 2018) using an Electrical Lab on a Photovoltaic-Chip (ELoPvC)—for obvious reasons—provide only a partial insight into the mechanisms and control of pollen tube growth (the co-variates such as the growth rate were not measured at this stage due to the extreme difficulty of such a simultaneous measurement; at least for a ELoPvC—a photovoltaic system, a bright-field microscope cannot be used as the light-induced EMF would exceed the measured signal by many orders of magnitude). Nonetheless, the idea of criticality that occurs in a growing plant cell appears to be in line with the detailed explanations that can be found in the literature, which combine growth rate oscillations with synchronised ion fluxes (see Fig. 2D in Damineli et al. 2017), where the extracellular H^+^ flux and growth rate curve almost overlap. Summarising, self-organised criticality is a mechanism through which open systems achieve a self-organised statistically stationary state in which they undergo a non-equilibrium phase transition.

The biotic or abiotic and endo- or exogenous factors that promote plant growth always have a very special, usually well-defined value for their tuning parameter. These parameters are either internal or external or even sometimes both. The pH and temperature are two of the most influential. The latter two, in the case of expansive growth, acquire optimum values for extreme growth (Pietruszka 2019). Then, the next question arises of whether the internal state of a system (evolving cell) is also characterised by a critical value of any single parameter for optimum growth. Such a single-parameter (*β*) statistic that can deliver a critical value at the optimum is presented in this work.

As an aside, the noise colour, where noise has been incorporated into the carrying capacity, was indicated by the spectral exponent (*β*) and included white (*β* = 0.0), red (*β* = 1.0), brown (*β* = 2.0), and black (*β* ≥ 3.0) noise; the currently used nomenclature is *β* = 1—pink or flicker noise (Johnson 1925) and *β* = 2—Brown noise. The time series is uncorrelated for low values of *β*; if 0.5 < *β* < 1, then there are positive correlations present in the time series as there are larger fluctuations on longer time-scales than expected by chance (Hardstone et al. 2012).

Hence, in what follows, we will explore and verify the hypothesis that criticality is a mechanism through which open systems such as plant cells achieve a self-organised stationary state. To test this hypothesis, we calculated the critical *β* exponents for two different plant species in normal and perturbed states. In addition to the plants that were studied, a droplet of human peripheral blood was also measured. The prediction to be tested was to verify whether *β* = 1 in optimal conditions.

## Materials and methods

**Fig. 1:**
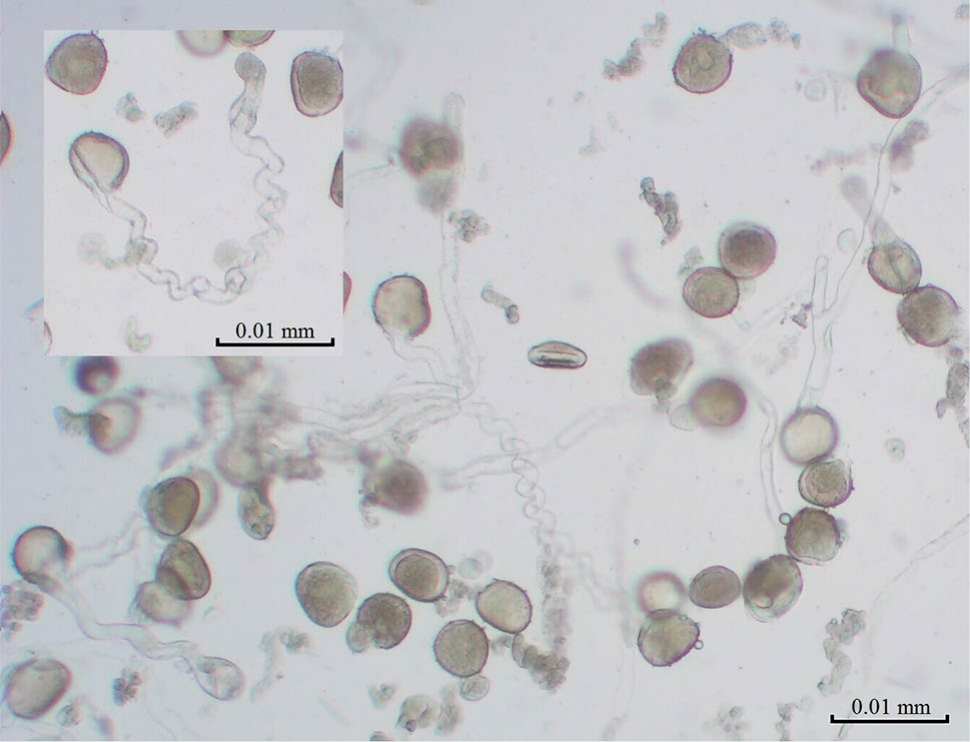
Photomicrograph of elongating pollen tubes of *Hyacinthus orientalis* L. Note the helical mode of growth (inset)

The measurements were performed *in extenso* on an elongating pollen tube (Fig. 1) of *Hyacinthus orientalis* L. and *Nicotiana tabacum* L. using an ELoPvC, which enabled us to observe extracellular ion fluxes in a single time series (Figure S1; compare lower inset with Fig. 1d in Cocchi et al. 2017 and Fig. 6 in Bak et al. 1988). The main advantage of this new experimental method is that the system being measured remains intact (grows freely) during the entire measurement and the response of the system is not disturbed by the measuring device as it is with electrode injection or incidental external or local electric and magnetic fields. This enabled us to distinguish “normal” growth, which corresponds with the control conditions, from a perturbed growth, which is equivalent to stress conditions or drug treatment, on a clear measuring platform. In the presented work, by “normal”, we mean in isotonic conditions at room (or physiological) temperature, while all of the other conditions were treated as perturbations. Both the normal and perturbed states can be further defined by researchers.

*Hyacinthus orientalis* L. was grown under controlled conditions at about 25 °C, 32–36% humidity and high insolation. Fresh pollen grains that had been collected from five flowers were immersed in a 2 ml Eppendorf tube containing 0.5 ml of a liquid germination medium (10% sucrose, 10 mg/l H_3_BO_3_, 300 mg/l Ca(NO_3_)_2_, 100 mg/l KNO_3_, and 200 mg/l MgSO_4_; pH 6.22 at 23.4 °C). The pollen grains were pre-incubated on an ELPIN + type 357 water bath shaker at a speed of 130 rpm for 1.5 h in the dark at room temperature. Research was carried out for several variants: hypertonic stress (25 mM NaCl, pH 5.20 at 21.6 °C), hypotonic stress (distilled water, pH 5.80 at 22.6 °C, 1:2 (v/v) dilution of the germination medium), IAA (4 mg/l, pH 6.00 at 22.6 °C), 2,4-D (100 mg/l, pH 3.12 at 21.2 °C), cold conditions (10–16 °C), and a control (only the germination medium).

The data for tobacco (*Nicotiana tabacum* L., clade: Asterids) were obtained from pollens that had been collected from flowering plants and had remained frozen since mid-August–mid-September (Oriental Samsun tobacco, Institute of Cultivation, fertilisation and Soil Science, National Research Institute, Puławy, Poland). The remaining experimental procedure was the same as for hyacinth for control but at different temperatures.

Measurements, which were performed using a CC-105 conductivity metre, revealed that the conductance of a medium at a level of about 0.40 μS/cm at close to 25 °C was required to ensure the proper (electrolytic) conditions during the measurements. The experiments were carried out on a single or multiple pollen tube(s) (Fig. 1). A single pollen with an elongating tube was selected and observed under a bright-field light microscope (Motic Microscope RED233). The selected pollen (or group of pollens), which was contained in a 20–40 μl germination medium, was transferred onto a photovoltaic semiconductor (n–p, phosphorus–boron, junction on Si crystal) plate (for more details, see Pietruszka et al. 2018) which was located in a grounded Faraday cage. After the transfer, the system was stabilised and the Tektronix DMM 4040 6–1/2 Digit Precision Multimeter voltmeter was calibrated after the deposition of the pollen onto a semiconducting plate; the entire process took about 15 min. Data were directly collected onto an external memory drive; 12 time series were collected per treatment. Each measurement was conducted in the dark for 20 min at a 4.1 Hz sampling (fulfilling the Nyquist sampling criterion, because the probed oscillation rate *f* can be assumed to be roughly *f* = 0.025 Hz for the long-period ion dynamics of a growing pollen tube; an average period of oscillation equals *T* = 39.1 ± 17.6 s (Hemelryck et al. 2018)). Welch vs high-pass/low-pass filters were used in Fourier analysis. The approximate external conditions at the time of the measurements were set to about 25 °C and 23–26% humidity in temperature range of 14–31 °C.

The final measurements were performed on the peripheral blood of *Homo sapiens* (female, 32), which had been taken from a finger immediately before the experiment, using an ELoPvC. Under sterile conditions, 40 µl of blood was obtained from the finger and was diluted 1:1 in 0.9% sodium chloride (NaCl). Then, 40 µl of the blood electrolytic solution was downloaded and transferred onto a photovoltaic semiconductor plate. The system was immediately stabilised for about 10 min. Each measurement was conducted in the dark for 20 min at a 4.1 Hz sampling. The external conditions were a temperature range of 27–39 °C and 33–43% humidity.

For further detail concerning the measurement principles, instruments, features of a crystalline solar cell, electromotive force (EMF) measurements, or the ion fluctuation measurements, see our recent paper (Pietruszka et al. 2018).

## Results

**Fig. 2:**
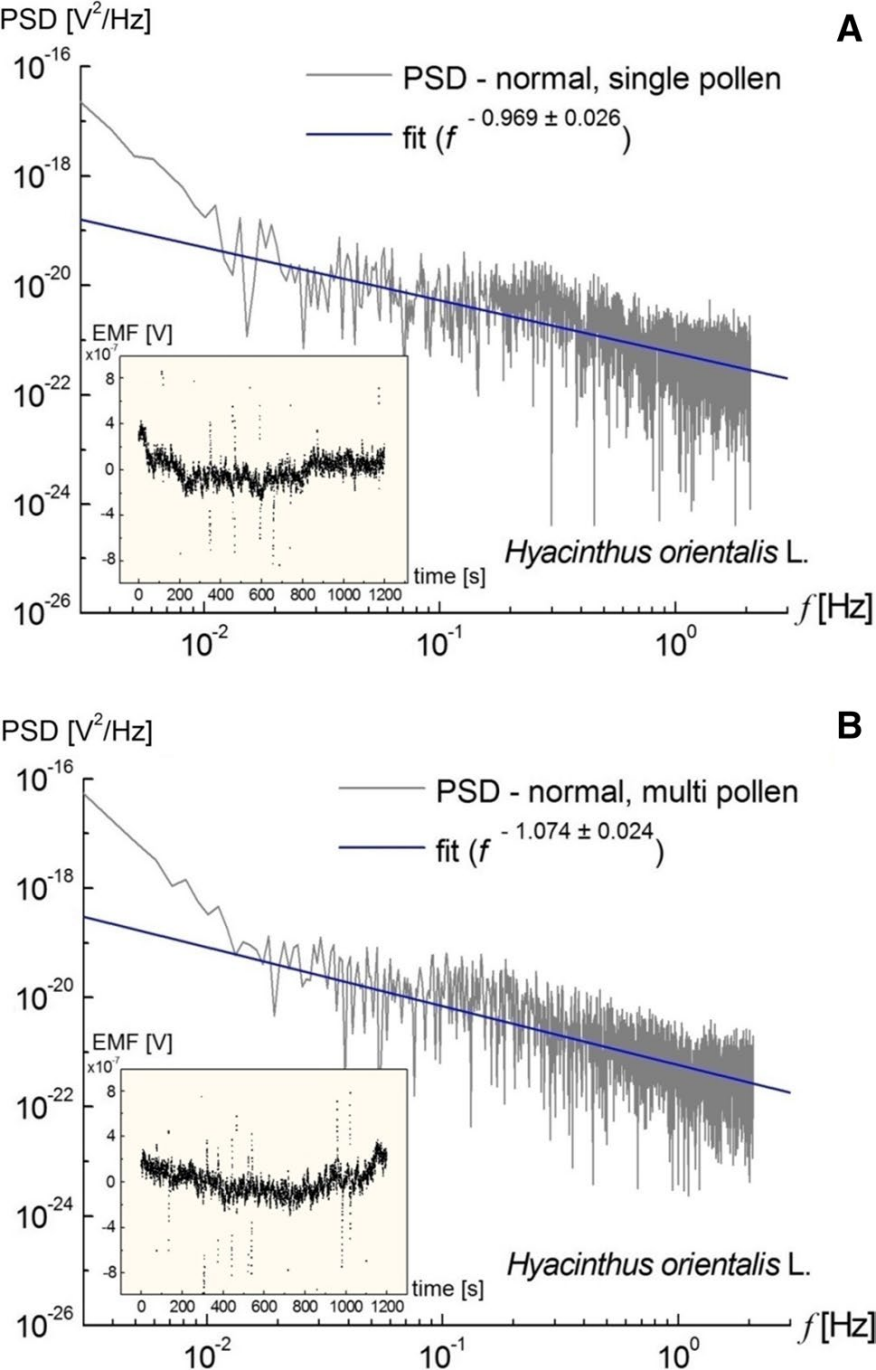
**a** Representative power spectral density (PSD) as a function of the frequency that was calculated for the EMF (volts) that were induced by the ionic fluxes of a single pollen of *Hyacinthus orientalis* L. (isotonic conditions, ~ 24 ± 0.5 °C) in double-logarithmic coordinates. The PSD, which was calculated using the Welch method and shadowed by the results of the discrete Fourier transform (light grey), manifested a 1/*f*^*β*^ noise behaviour for about 3 decades of frequencies (a feature that is highly characteristic for the membrane ion channels). The actual fit, which resulted in a power law (*β* = 0.969 ± 0.026), is indicated by the blue line. **b** The PSD as a function of the frequency that was calculated for a group of pollen grains of *Hyacinthus orientalis* L. (isotonic conditions, ~ 25.3 ± 0.5 °C). The actual fit (*β* = 1.074 ± 0.024) is indicated by the blue line. The spectral density was derived from a single representative time series (inset), which displayed clear oscillations for 20 min. Note that multiple tubes essentially behave as a single tube in regard to *β*

The ELoPvC experiments were performed on the elongating *Hyacinthus orientalis* L. pollen tube(s) and for *Nicotiana tabacum* L. at different temperatures. The results from the multiple time series (Figure S1) that were measured are presented in Fig. 2, Figures S2–S3, and are summarised in Supplementary Tables S1–S10. The power spectral density (PSD) as a function of frequency $P\left( {\upomega } \right) = \left| {x\left( {\upomega } \right)} \right|^{2}$ where $x\left( {\upomega } \right) = \mathop {\lim }\limits_{T \to \infty } \mathop \smallint \limits_{0}^{T} x\left( t \right){\text{e}}^{ - i\omega t} {\text{d}}t$ and ${\upomega } = 2{\uppi }f$ was calculated for the electromotive force, EMF $\left( { = x\left( t \right)} \right)$ that was induced by the incoming or outgoing ionic fluxes of a single or multiple pollen(s) of *Hyacinthus orientalis* L. in normal (isotonic, unperturbed) or perturbed conditions (indole-3-acetic acid (IAA) treatment, 2,4-dichlorophenoxyacetic acid (2,4-D) treatment, hypertonic stress, hypotonic stress, cold). The Welch method (or a 1.37 Hz low-pass and 0.0025 Hz high-pass filter), which is typically used to reduce noise in the estimated power spectra in exchange for reducing the frequency resolution (Proakis and Monolakis 2007), was used to deliver the slope *β* of the PSD that was obtained using the Fourier analysis (Microcal Origin software). Note that there are limitations and assumptions being made concerning the linear fit to the PSD, since the results are coherent and bring an interesting and novel perspective to the ion exchange of rapidly growing cells. A single value of *β* (PSD ~ 1/*f*^*β*^ where *f* stands for frequency) was obtained for each (counting 5000 time points) 20 min measurement (Fig. 2, Figures S2–S3); for multiple pollens,* N* = 12 was the number of repetitions of an experiment of a given type to obtain the average value of < *β* > . This value was compared with the value of the averaged *β* that had been obtained for normal growth (Fig. 2) using a paired *t* test (Table S1), ANOVA tests (Table S2–S8), and *t* tests for one population mean (see Supplementary Information). Intriguingly, only the normal growth of pollens brought a *β* = *β*_c_ = 1 result that was within the experimental error. The data for all of the remaining treatments delivered *β* ≠ 1, *β* < 1 (Table S1–S8). Similar data for the *β* exponent were collected for *Nicotiana tabacum* L. (Figure S3), except treatments.

For all of the stress conditions that were tested, hypertonic stress, hypotonic stress, and cold, the ion flux flow was inhibited compared to the control conditions (Table S1). The strongest effect of a stress treatment was observed for cold stress (corresponding to the slowest elongation rate of the pollens), which resulted in a decrease in the *β* that was delivered (up to 0.8035 ± 0.04). In addition, hypotonic and hypertonic stress perturbed the ion flux—*β* equal to 0.8747 ± 0.09 and 0.9517 ± 0.06, respectively, compared to the control conditions—0.9985 ± 0.08. Treatment of the pollens with auxin also resulted in a decrease in *β* and similar results were obtained for both natural (IAA) and synthetic (2,4-D) auxin—0.8522 ± 0.07 and 0.8537 ± 0.05, respectively. What seems to be important for the analysis is the fact that the normal/cold difference in the value of *β* delivered an “extremely statistically significant” result. Moreover, the use of the growth factors IAA and 2,4-D (natural and artificial auxin, respectively) delivered a difference in the ANOVA with Tukey HSD test that was not statistically significant, which fulfilled our predictions as they should not differ critically, at least at this level of refinement.

Furthermore, we conducted the *t* test for the normal state mean of the *β* exponent for *Hyacinthus orientalis* L. (see the paragraph in Supplementary Materials for details). We concluded that the null hypothesis *H*_0_: < *β* > _pop_ = 1 was not rejected. Therefore, there was not enough evidence to claim that the population mean *β*_pop_ was different than 1 at the 0.001 significance level. The 99.9% confidence interval was 0.896 ≪ *β* > _pop_ < 1.101 (Figure S4).

In addition, we conducted another *t* test for the normal state mean of the *β* exponent for *Nicotiana tabacum* L. (Supplementary Materials). We concluded that the null hypothesis *H*_0_: < *β* > _pop_ = 1 was also not rejected in this case. Therefore, there was not enough evidence to claim that the population mean *β*_pop_ was different than 1 at the 0.001 significance level. The 99.9% confidence interval was 0.779 ≪ *β* > _pop_ < 1.189 (Figure S5).

**Fig. 3:**
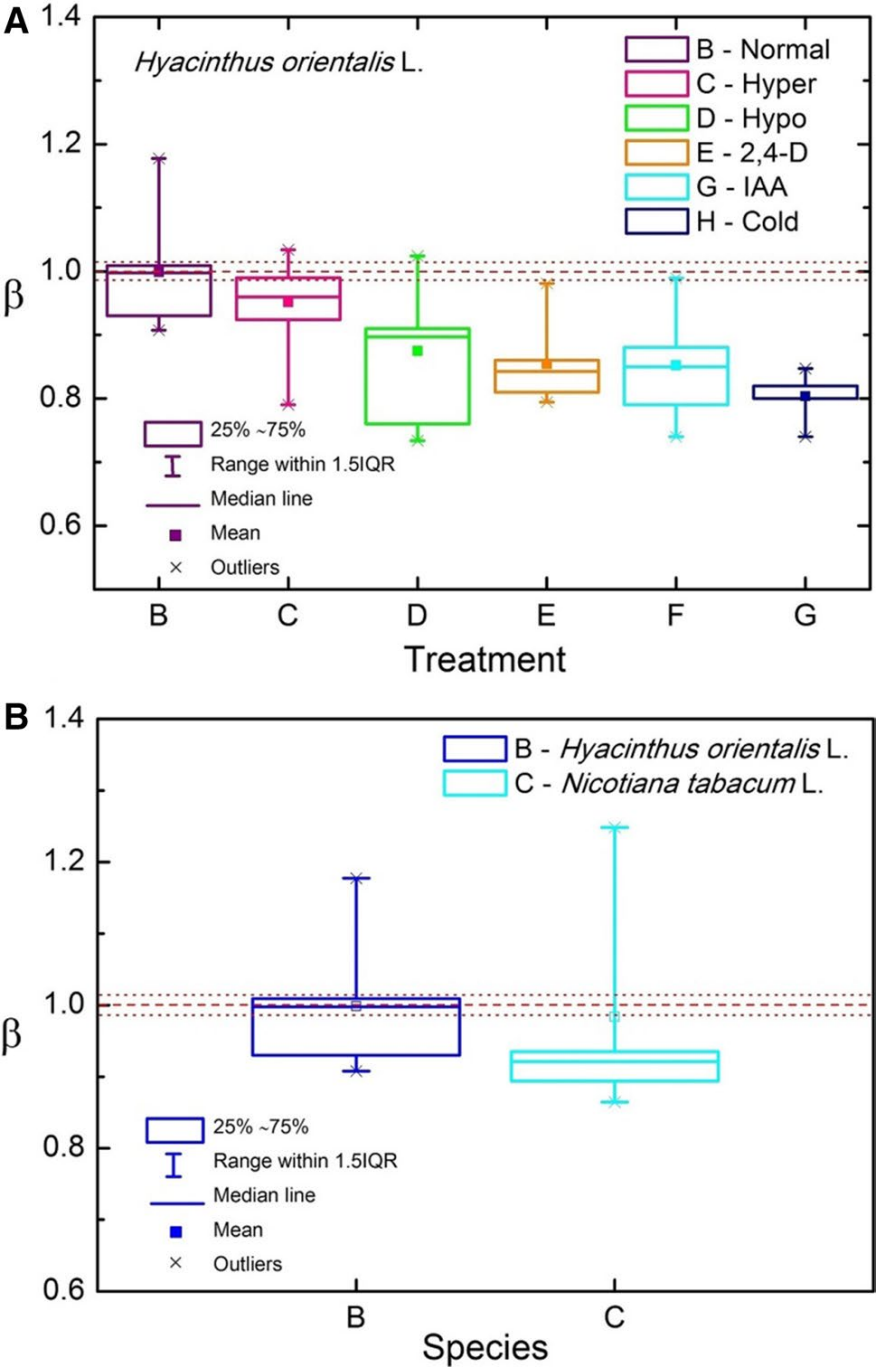
**a** Average value (mean), median, and inter-quartile range for the spectral exponent *β* for the different perturbations (normal state) indicated in the box chart. The *β* = 1 (dashed line) and the 95% confidence interval (dotted lines) for hyacinth are indicated in the chart. **b** The same as in A but for the two different species in normal state indicated in the chart. The average (of 12 samples) temperatures of measurements for *Hyacinthus orientalis* L.: 25.0 ± 0.5 °C and for *Nicotiana tabacum* L.: 23.9 ± 0.5 °C

Additionally, to be on the safe side, the treatments were compared using the ANOVA test for multiple pollens of *Hyacinthus orientalis* L. (input data—Table S2); descriptive statistics—Table S3; one-way ANOVA of six independent treatments—Table S4; Tukey HSD results—Table S5; Scheffé results—Table S6; Bonferroni and Holm results: pairs compared simultaneously—Table S7; Bonferroni and Holm results: only pairs relative to a normal state compared simultaneously—Table S8. All of the tests confirmed the above claims and the results are presented in the boxplot-style in Fig. 3a with a line indicating the critical value of *β* = 1.

**Fig. 4:**
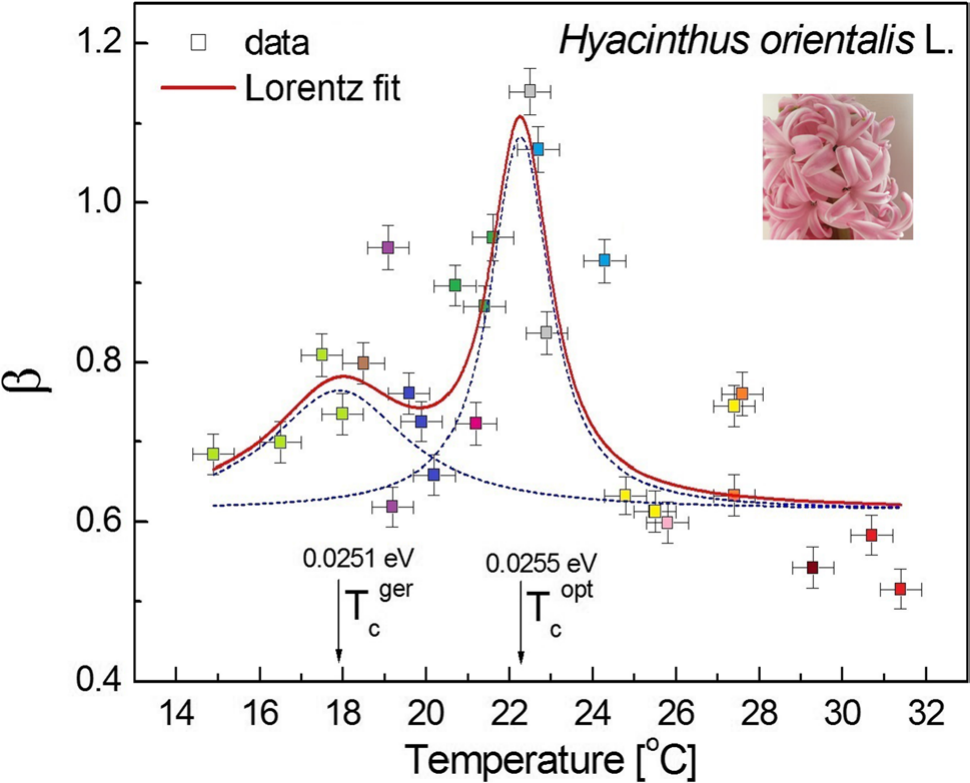
Resonance curve of the spectral exponent *β* as a function of temperature (control parameter) for *Hyacinthus orientalis* L. pollen tubes. The critical temperatures *T*_c_^ger^ = 17.91 ± 0.25 °C (half-width 3.94 ± 0.98 °C, peak area = 0.93 ± 0.22) and *T*_c_^opt^ = 22.27 ± 0.05 °C (half-width 1.65 ± 0.21 °C, peak area = 1.25 ± 0.1). The corresponding energy (in electron volts) is indicated in the chart, while *β*_c_^ger^ = 0.93 ± 0.22 and *β*_c_^opt^ = 1.25 ± 0.11; *β*-offset = 0.61. The resonance peak corresponds to the critical fluctuations in the system. The different colours of the interlaced data correspond to the different series of measurements over the subsequent days of the experiment. The solid red line corresponds to the Lorentz fit (*R*^2^ = 0.64), which is interpolated by the B-spline (Microcal Origin 6.0). The temperature was measured at 20 min measurement intervals with an accuracy of ± 0.5 °C. The measurement errors are represented by the drone-like objects in the plot. Compare with Figure S6 for control conditions

A similar plot is presented in Fig. 3b for the different plant species. The average measurement temperatures for *Hyacinthus orientalis* L. (25.0 ± 2.1 °C) were considerably higher (about 1 °C) than the mean for *Nicotiana tabacum* L. (23.9 ± 0.6 °C). The data for the hyacinth were obtained during February–April. It turned out that temperature is a critical parameter for this kind of measurement (compare with Fig. 4—the half-width of 2.13 ± 0.62 ≈ 2 °C).

The PSD for control conditions for germination medium and sodium chloride solution are presented in Figure S6. Note the low value of spectral exponent *β* that is closer to the white noise signal.

## The temperature dependence of the spectral exponent

### Vital episodes from cell life on the verge of criticality

**Fig. 5:**
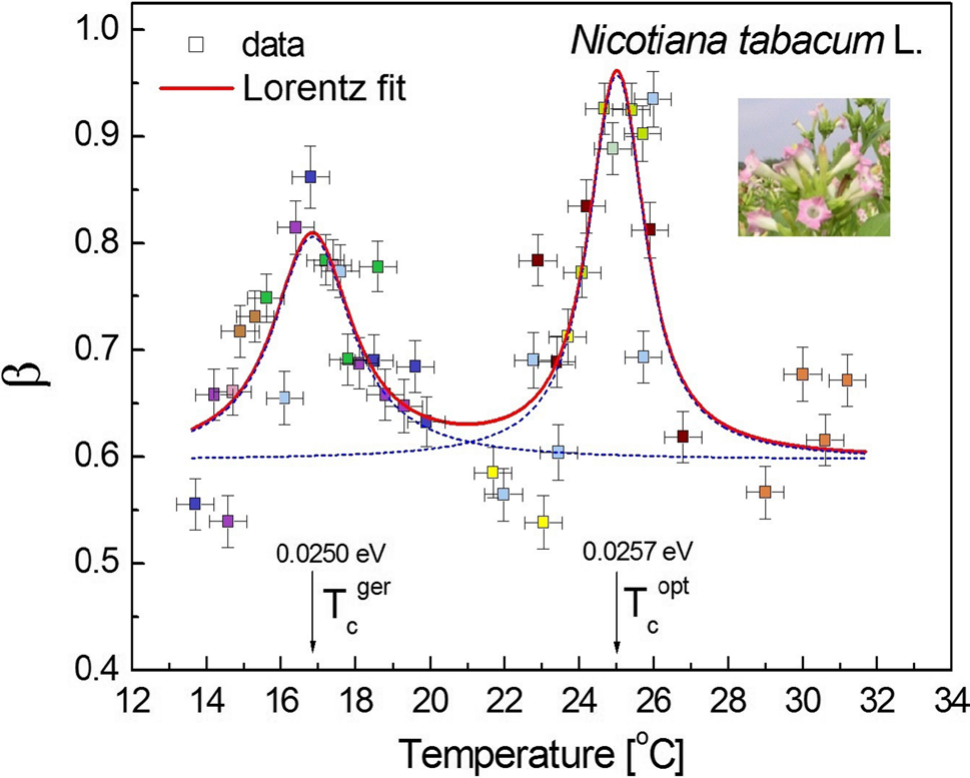
Resonance curve of the spectral exponent *β* as a function of temperature (control parameter) for *Nicotiana tabacum* L. (Oriental Samsun) pollen tubes. The critical temperatures *T*_c_^ger^ = 16.85 ± 0.08 °C (half-width 2.46 ± 0.35 °C, peak area = 0.83 ± 0.11) and *T*_c_^opt^ = 25.02 ± 0.03 °C (half-width 1.71 ± 0.18 °C, peak area = 1.01 ± 0.08). The corresponding energies are indicated in the chart, while *β*_c_^ger^ = 0.83 ± 0.11 and *β*_c_^opt^ = 1.00 ± 0.08; *β*-offset = 0.6. The solid red line corresponds to the Lorentz fit (*R*^2^ = 0.72), which is connected (smoothed) by the B-spline

The influence of temperature on the *β* exponent is presented in Figs. 4 and 5 (see also Figures S7–S8 for raw data) where the Lorentz resonances localise the two critical temperatures: germination (see Rosbakh and Poschlod (2016) and opt 2010) and optimum growth. It seems that these important results dispel doubts that might arise for the interpretation that is proposed throughout this paper. Normal growth acquires a critical value of *β* close to 1 and this is not accidental (optimal growth coincides with power-law voltage fluctuations). The maxima in the resonance curve seem to correspond to critical fluctuations in the system. On the other hand, they also correspond to the situation in which the lowest frequencies of the oscillations dominated and reached the (coherent) dynamic state of equilibrium during growth. There, the high-frequency modes were reduced and the dissipation of energy was minimised. This is, however, what has been already recognised in longitudinal and transverse pollen tube oscillations (Haduch-Sendecka et al. 2014). Even though an external variable (temperature) that could have been treated as a “control parameter” of this process was changed, some recent data indicate (Pietruszka 2019) that it is a change of the chemical potential, which is dependent on temperature, which induces criticality in the investigated system (note that the chemical potential implicitly and explicitly enter the definition of pH:${\text{pH}} = {\text{pH}}\left( {\mu_{{H^{ + } }} \left( T \right), T} \right)$).

### Temperature-induced sudden onset of coherent action in pollens—Lorentz resonances

For illustration, consider the system of *Hyacinthus orientalis* L. (or *Nicotiana tabacum* L.) pollens, which consists of a collection of ion-active cells (pollen tube tips) that are embedded in an electrolytic medium and electromagnetically isolated using an aluminium mirror, a Faraday cage (Pietruszka et al. 2018). An external energy source (thermostat at temperature *T*) is used to excite or “pump” the ions in the elongating pollen tubes out of their ground state (at a low temperature). Each pollen tube tip can be thought of as a little antenna (Strogatz 1994) that radiates energy in the form of the appropriate (H^+^, K^+^, Cl^−^, and Ca^2+^) ion fluxes. When the “pumping” is relatively weak (low temperature), the “laser” (a system of pollen tubes) acts like an ordinary lamp; the excited tubes oscillate independently of one another and emit randomly phased ionic flux waves. Now, suppose that we increase the strength of the pumping (by raising the temperature). At first, nothing different happens, but then suddenly, when the pump strength (ambient temperature equivalent to the supplied energy) exceeds a certain threshold (critical temperature), the pollen ion fluxes begin to oscillate in a phase—the “lamp” has turned into a “laser”. Now, the millions of small “antennas” (ion channels) act like one giant antenna and produce a beam of “radiation” (polarised wave) that is much more coherent and intense than the one produced below the critical threshold (temperature).

This sudden onset of coherence is remarkable, considering the fact that the pollen ion fluxes are being excited by temperature completely at random. Hence, the process is self-organising; the coherence develops because of the cooperative interaction among the ion fluxes of the pollen tubes themselves. The critical “pumping” by temperature is clearly seen in Figs. 4 and 5; it can correspond to a transition that marks the onset of synchronous (i.e., correlated) behaviour. This process coincides with the temperature (energy) of optimum growth. The Lorentz peaks can be interpreted as “coherent states” of the “laser action” of an evolving plant cell.

Finally, we can ask, what is the difference between ordinary growth and extreme (optimum, fast) growth at this level of refinement? It seems that it is the ion coherent states that cause the diminished or even infinitesimally small energy dissipation in a process that we can call an orchestrated instability or SOC at optimum growth.

### Life at the ridge of criticality

**Fig. 6:**
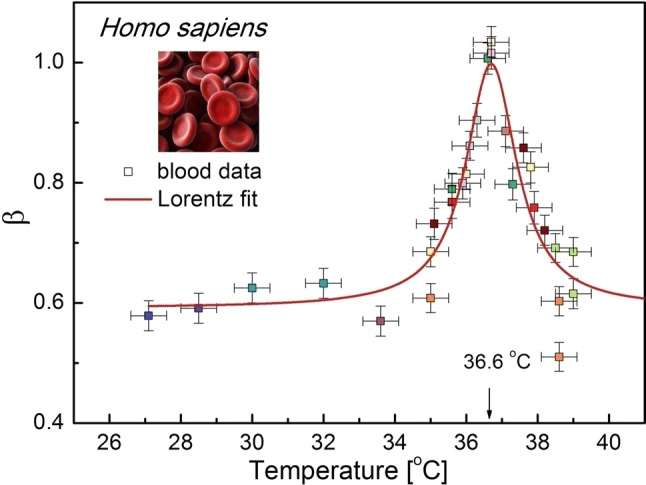
Resonance curve of the spectral exponent *β* as a function of temperature for *Homo sapiens* blood. The optimum temperature *T*_c_ = 36.69 ± 0.06 °C (half-width 1.71 ± 0.24 °C, peak area = 0.98 ± 0.20. The solid red line corresponds to the Lorentz fit (*R*^2^ = 0.85), which is connected (smoothed) by the B-spline. The temperature was measured at 20 min measurement intervals with an accuracy of ± 0.5 °C. The measurement errors are represented by the drone-like objects in the plot. Note that at a physiological temperature of about 36.6 °C, the spectral signature *β* equals 1

Here, we show that the temperature dependence of *β* is not exclusive to pollen tubes. The vital role of temperature for cell life is also especially visible in preliminary results presented in Fig. 6 in which the pronounced peak at a physiological temperature of 36.6 °C for a human being appears (also compare with Fig. 1 in Pietruszka and Lipowczan (2017) for water anomalies and physiological temperature). The resonance-like curve showed in Fig. 6 shows criticality at the "optimal" temperature. The latter may indicate that a “healthy” (i.e., normal) cell evolves along a *β* = 1 path. This result strikingly suggests that a living system (here: a human being) is “prepared” (encoded) by corresponding evolutionary mechanisms to live along this “thin red line” where it permanently attempts to “sustain criticality” (Longo and Montévil 2011) to achieve the optimum performance. A temperature that is slightly lower or higher than 36.6 °C must be corrected in order for the system to operate most effectively. This is, however, in accord to Fig. 6 where a very special, i.e., critical temperature was identified. In accordance with our intuition, a system that is too far from this attractor can never return to these optimum conditions (Wolf et al. 1985). However, this fact reveals the existence of a very sophisticated and accurate fine tuning in systems that are endowed with life.

### Dominant Lyapunov exponent and Pearson correlation coefficient

We also estimated the largest Lyapunov exponent (LLE). The Lyapunov exponent is by definition the rate of the exponential separation with time of initially close trajectories. The Lyapunov exponent describes the speed of convergence or divergence of trajectories in each dimension of the attractor. Since all *λ* exponents were less than zero (Table S10 and Figure S9), this indicated a fixed-point dynamics or periodic volatility of the investigated system [With *λ* < 0, the system tends to one stable state (e.g., diffusing systems, such as a damped pendulum) or a periodically variable state—oscillations]. Also, due to the regular distribution of the LLE, we calculated a correlation coefficient. The Pearson correlation coefficient is used to measure the strength of a linear association between two variables, where the value *r* = 1 means a perfect positive correlation. We used this test to find out whether the *β* spectral exponent and LLE are correlated (normalised data). It turned out that *r* = 0.9198 ≈ 0.92, meaning that the correlation was high. According to the very definition from the introduction: if 0.5 < *β* < 1, then there are positive correlations present in the time series, and with *λ* < 0, the system oscillates.

## Discussion

Although a steady rise in entropy is a basic principle in the universe, on the other hand, a high degree of organisation and the fight against entropy are the basic laws of life. Many extended dissipative dynamic systems evolve into structures that have long-range spatial or temporal correlations with the 1/*f* power spectrum (Tang and Bak 1988). It has been suggested that this behaviour may be caused by the self-organisation of systems into a “critical state”. It appears that a similar hypothesis can be applied to a growing plant cell, here a freely evolving pollen tube.

In this work, we sought evidence of the high level of complexity in the electrical dynamics of plant cell(s) signalling with unprecedented accuracy at the microvolt level (corresponding to the energy of a micro electron volt [μeV], i.e., 1 × 10^–6^ × 1.60217662 × 10^–19^ J ≈ 10^–25^ J). From the “EEG-like data” analyses of an intact growing *Hyacinthus orientalis* L., *Nicotiana tabacum* L. pollen tube, we showed consistent evidence of critical dynamics and long-range correlations in the electrical time series. Furthermore, we found that the dynamic complexity of the electrical signals is affected by the physiological conditions of a plant cell, which changed intensively when the plant was stressed. We observed that electrical spikes arose following a power-law distribution in the frequency domain, which is indicative of SOC, a phenomenon that has already been recognised in physics and neurobiology due to the metaphorical visualisation of the model of a “sand pile” on which new sand grains were slowly sprinkled in order to cause “avalanches” (Bak et al. 1988). If the “slope” of a system is somehow kept larger than the “critical slope”, there will be a continuous “spontaneous flow”. A similar situation can probably be encountered in the ionic channel (coupled to growth) where the ion flux flow occurs after reaching a critical value at the gate and opening the gate for specific ions. Apparently, this feature can be recognised by our method and can be interpreted as SOC that is evoked during pollen tube growth. In this context, the ionic SOC mechanism could be responsible for the long sought after mechanism of temporal and spatial pollen tube oscillations.

The 1/*f* noise is the dynamic response of a sand pile to small random perturbations (Tang and Bak 1988). Therefore, a specific explanation for the enigmatic pollen tube oscillations may emerge directly from SOC, since the membrane ion diffusion processes, which are triggered by the level of the chemical potential, can be treated as the possible origin of the 1/*f* noise. The periodicity and synchronisation in growing cells can possibly be attributed to cyclic “avalanches” like in the sand pile model that correspond to dynamic “phase transitions” (Hesse and Gross 2014; Tang and Bak 1988) or “state transitions” (Pietruszka 2019) in this highly non-equilibrium system. The control parameter is no longer controlled externally, but by the evolving system itself—the chemical potential may be the best candidate (Van der Marel 2004; Pietruszka 2019). It has been suggested (Longo and Montévil 2011) that biological systems share two key properties of phase transitions: a change of macroscopic behaviour (such as the extreme growth in pollen tubes) and the coherence of the system at a critical point, which will be discussed further. A recurrent idea in the study of complex systems is that optimal information processing is to be found near-phase transitions. However, this hypothesis has a few concrete realisations where a standard and biologically relevant quantity is optimised at criticality (Kinouchi and Copelli 2006).

Since the explanation of the 1/*f* noise that is caused by SOC was given (Bak et al. 1988), many complex systems have displayed self-organised critical states that are characterised by such a frequency scaling of the power spectra (Dutta and Horn 1981; Schroeder 1991; Hesse and Gross 2014; Markovic and Gros 2014). Others have shown 1/*f* noise in single ion channels, e.g., Liebovitch and Sullivan (1987). The difference between the models and experimental results on innate matter is important, because evidence for self-organised criticality is considerably scarcer in real systems than in models.

Cells are empirically revealing themselves to be inherently dynamic, self-organising systems that respond stochastically and nonlinearly to environmental stimuli (Nicholson 2019). Considering a plant cell, 1/*f* scaling is interesting, because it reveals long-lasting correlations or long-range spatial coherence in a growing cell system, which is similar to the behaviour close to critical points (Bédard et al. 2006). In physics, criticality is defined as a specific type of activity that is observed when a system undergoes a phase transition (Hesse and Gross 2014). If a system has a continuous phase transition, then it can reside at the transition point between two phases on the edge (critical state) between two qualitatively different types of behaviour. Usually, at criticality, the symmetries of a system such as ordered and (less/dis)ordered are broken. The general idea of systems tuning themselves to criticality through active extended processes is known as SOC. A similar mechanism has been proposed (Pietruszka 2019) concerning the dynamic “state transitions” (from an ordered to a disordered state, from a stressed to a relaxed state, and from a “covalent bonds state” to a “disruption of covalent bonds state”), which can occur in the peripheral cell wall in systems that are far from equilibrium. Indeed, a hidden variable, a control parameter for this decentralised, though spatially coherent process, was proposed to be the chemical potential (ibid.) of a specific ion species. Similar to SOC, the control parameter is no longer controlled externally, but by the system itself. The interpretation of the synchronised oscillations observed in single pollen tubes (Pietruszka et al. 2018) as "phase transitions", where the system itself tunes a control parameter, may have great potential. However, an alternative framework exists to explain transitions into an oscillatory state that is the classical bifurcations that give rise to oscillations in a deterministic dynamical system (Cocchi et al. 2017). The transition from steady state to cyclic behaviour due to strong interactions is called a bifurcation; the strength of the interaction is called a control parameter and the point at which the bifurcation occurs is denoted the critical point (ibid.). This mechanism is an alternative to SOC as it would generate a characteristic frequency yet with complex transitions depending on the dynamics of the so-called control parameter. Still, there could be an internal control that would place the system close to the bifurcation point (the critical value), mimicking the complex dynamics attributed to SOC.

“*Plus ultra*” We also suggest that ion transport across the cell membrane may involve quantum coherence at an ambient temperature (Collini et al. 2007; Marais et al. 2018) that is facilitated by the coloured noise (see inset in Figure S1A and compare it with Fig. 2 in Engel et al. 2007) that is generated by the molecules that constitute the ionic channels. This contrasts with the long-held view that long-range quantum coherence between molecules cannot be sustained in complex biological systems. In our case, the efficiency of the ionic energy (~ μeV) transfer was dependent on the temperature (Table S1) and other environmental cues (Fig. 2 and Figure S2). It turned out that a very special value of coloured (pink) noise with *β* = *β*_c_ = 1 was selected by a system for optimum growth. However, the source of the coloured noise is the vibrations of the molecular structures (here: ionic channels) (Al-Khalili and McFadden 2014; Marais et al. 2018) which maintain coherence in the cell membrane beyond the diffusive growth. The PSD that was obtained revealed a 1/*f* pink noise, which reciprocated the randomly alternating open/close states of the channels. This stochastic phenomenon is known as channel noise, which can be directly related to the channel conductance fluctuations. The *β* value that was obtained differed drastically for normal and cold conditions—hence, apparently, the Goldilocks Zone was found where the molecular vibrations that cause the conductance fluctuations of a cellular membrane are precisely those that are required to modulate the ion current flowing into a biological cell. The signal that was obtained in these subtle measurements (Figure S1), which were performed at the microvolt (μV) level, resembles the quantum rumble that was observed by the Fleming group (Fig. 4.7 in Al-Khalili and McFadden 2014) though at a different time scale. This is also in accordance with the view that the “wet and noisy” environment of a living cell may assist quantum dynamics and maintain quantum coherence rather than destroying it (Mohseni et al. 2008; Plenio and Huelga 2008; Caruso et al. 2009). Obviously, this is only a speculation, but the possible consequences of such coherence at a specific temperature (energy) are extraordinary and would make 1/*f* systems insensitive to any disorder in the distribution of energy.

1/*f* fluctuations were first observed in electronic devices (Schottky 1926), and now, they are ubiquitous in nature. To distil the essence of the SOC phenomenon for living cells, more experiments are required. However, further exploration demands extreme (stable) experimental conditions (especially temperature) and even more sophisticated measuring devices (an 8½-digit voltmeter), which were beyond our reach. Only then, can an important subject concerning external, environmental cues at the organism level (Souza et al. 2017) and many others that are mentioned here be properly addressed. Moreover, all future experiments should be performed with caution (Yakushenko et al. 2014) to avoid any artefacts that might originate from the chemistry of the nanoparticles that are used in the solute-semiconductor interface. Finally, some critical distinctions have to be made, especially when considering multiple pollen tubes. In these measurements, there is no a priori reason to recover the characteristic time scale (unless all tubes are synchronised in phase) like in our previous paper (Pietruszka et al., 2018). Furthermore, considering the entire ensemble as a SOC system is a different matter than the regulation of ion fluxes across the tip membrane of a single tube. Nonetheless, probing criticality in external ionic fluxes corresponds to the shadows observed in the inverse Plato's cave allegory and can provide information about participating cells.

Note that 1/*f* frequency scaling for normal systems indicates a long-range correlation, i.e., although system dynamics is not random, it is not necessarily an SOC system (Bédard et al. 2006; Gao et al. 2006). While for self-organised critical systems, one does expect power laws for the spatial and temporal correlations, the observation of power laws is not sufficient to claim criticality as also other mechanisms can lead to power laws (Marković and Gros 2014; Beggs and Timme 2012; Bonachela and Muñoz 2009). Even the detection and proper characterisation of power laws is complicated by the large fluctuations that occur in the tail of the distribution (Clauset et al. 2009).

1/*f* processes have been discovered in biological systems such as DNA sequences, human cognition, coordination, or posture (Preussner 2012). An important subclass of 1/*f* noise is those that have a long-range temporal correlation or long memory in a time series (Gao et al. 2006). The possibility that 1/*f* fluctuations are observed in many biological time series results from the fact that biological processes have many inputs that act on different time-scales is questioned. It is unlikely that the 1/*f* behaviour that is observed in many biological systems is only due to the fact that these systems are regulated by many different inputs that are acting on different time-scales (Hausdorf and Peng 1996). In our investigation, an experimentally based agreement was found with the concept of the self-organisation of living systems towards criticality at the 1/*f* “edge of chaos” (Ito and Gunji 1994). It is obvious that much of the data that is obtained in experiments on living systems is not subjected to extensive analysis to probe for critical behaviour and finite-size scaling (Preussner 2013). This raises the question (ibid.) of whether a concept such as SOC could be at work in a biological system where it cannot be probed for. However, it seems that our experimental technique enables enough dense high-resolution data for such extensive analysis to be obtained.

In this and our previous study, we used the ELoPvC technique, which enabled observation for the first time of the 1/*f* or even SOC behaviour in the ionic balance across the plasma membrane in a single time series in the growing pollen tubes of *Hyacinthus orientalis* L., *Nicotiana tabacum* L. under different conditions. We showed that the ELoPvC technique can be used to investigate the effect of a hormonal treatment (IAA, 2,4-D) or abiotic stress (hypertonic stress, hypotonic stress, and cold—resulting in the least growth) on intact single plant cells. Thus, it seems that ELoPvC is a promising technique that can be widely used in cell studies. By analysing the power spectral density function (PSD), we calculated the beta exponent from PSD ~ 1/*f*^*β*^ taking into account *β* = 1 as the SOC signature. In the experiments, only the normal growth of pollens delivered a *β* = *β*_c_ = 1 result that was within the experimental error. The data for all of the remaining treatments delivered *β* ≠ 1. Using a growing pollen tube as a suitable cell model, we examined the theoretical and applied issues that can expand the understanding of the electrophysiological dynamics in plant cells. Now, additional experiments using other plant model systems are required to confirm the versatility of the ELoPvC technique.

Moreover, it has to be stressed that the spectral exponent was the only parameter of interest that was calculated directly from the measurements. Since it significantly differs for different cues (Tables S1–S10), it provides a single-parameter statistical mechanics description of the complex ion-exchange dynamics that are observed in apically growing cells that is easy to use and interpret for further investigations. The presented solute–semiconductor interface technique can also be applied for more complex analyses including nano-volt measurements of cell growth using a high-resolution, fast CCD camera. Then, even the calculation of cross-correlations between those two (directly interrelated) simultaneous measurements can be imaged, thus resulting in a complementary picture of the relationship between the ion efflux and cell growth.

The 1/*f* signature is a characteristic statistical feature of transmembrane ionic transport processes. On the other hand, 1/*f* noise is a curious phenomenon, which is especially pronounced in far-from equilibrium systems. Virtually, all biophysical processes that underlie pollen tube growth—vesicular trafficking, cytoskeleton dynamics, intracellular electrokinetic (electro-osmotic) and chemi-hydrodynamic fluxes, and their reciprocal osmo- electro- and chemiphoretic interfacial transport phenomena, cell wall loosening, and extension—are, to some extent, non-equilibrium and can play a part in generating a flicker-noise signature. This aspect (although not fully addressed as yet) opens up new and rich perspectives for further research.

The drive to survive is a biological universal. According to Calvo et al. (2019), intelligent behaviour is usually recognised when individual organisms including plants change their behaviour to improve their probability of survival when met with fiercely competitive or adverse, real-world circumstances. This competitive behaviour was observed here as is visible in Figs. 4 and 5. The plot of the spectral exponent *β* as a function of temperature, which can be treated as a control parameter for *Nicotiana tabacum* L. and *Hyacinthus orientalis* L. pollen tubes, revealed a double resonance curve at critical temperatures. The maxima of the latter corresponded to the situation when the lowest frequencies of oscillations dominated and reached the correlated dynamic state of equilibrium, which was similar to the dynamic kinetic stability (DKS) state during growth that was reviewed in Pross and Pascal (2013). There, the high-frequency modes were reduced and the dissipation of energy was minimised (compare with Haduch-Sendecka et al. 2014), thus allowing for extreme growth. At criticality, the investigated system possibly proceeds along a “path of the least action” (Feynman and Hibbs 1965), which indicates that fertilisation and inheritance are the directed goals and are thus purposeful.

The presented approach, if successful, would estimate a characteristic distribution of fluctuation sizes by simply using the log–log slope of the Fourier power spectrum (*β*), which could potentially distinguish “healthy” from “unhealthy” growth. A *β* of value 1 would suggest that a system is in a self-organised critical state with a characteristic power-law distribution of fluctuations that support properties such as the long-range spatio-temporal correlations and self-tuning of the control parameter that is responsible for bifurcations in the dynamics.

The issue of criticality is attracting the attention of an increasing number of neurophysiologists (Lovecchio et al. 2012). It cannot be excluded that a critical exponent with a value of 1 might be also expected in the psychophysical domain. Whether the state of mind is quantum or classical has been a matter of debate since the coherent states that are represented by the Lorentz resonances were also believed to play a crucial role in the transition from quantum to classical (Zurek 2002). However, currently, the possible answer is that it positions itself at the border where *β* equals 1. It seems that SOC may be a generative mechanism for psychophysical laws (Kello et al. 2010) and let us interpret the brain as a source of ideal 1/*f* noise.

## Cell life—at the 1/f ridge of extended criticality?

Finally, “survival of the fittest”, a phrase that was coined by Herbert Spencer that originated from the early Darwinian evolutionary theory as a way of describing the mechanism of natural selection, which was controversial for its tautological character, can now be examined in regard to all of those systems that evolve at the ridge of criticality where the concept of ongoing or “extended criticality” (Longo and Montévil 2011, 2013) may apply. Moreover, the idea that biological time forms a two-dimensional entity (Bailly et al. 2001) in which one dimension is classical, while the other dimension is compacted on a circle, is in agreement with our observations. The first dimension is parameterised according to the line of real numbers, which are limited by fertilisation on the one hand and cell death on the other, while the second dimension is linked to an organism’s endogenous physiological rhythm, which was also observed in our experiments. Our results indicate that biological entities experience criticality over a non-zero time interval, which can be called the ridge of criticality (see Figure S11 for illustration), not only at a (critical) point along the line where the intended control parameter runs.

The basic idea behind the work of Kinouchi and Copelli (2006) is that in biological complex systems, the optimal information processing is found near-phase transitions, and that the efficiency of biologically relevant processes is optimised at criticality. This is, however, exactly what we found by probing the coexistence of temporal complexity and periodicity of living entities such as optimally evolving pollen tubes that preserve 1/*f* scaling.

## Conclusions

Our paper reports on the measurements of the electromotive force that is generated in vivo by plant cells individually and collectively, and describes a flicker-noise signature of the measured signals. The obtained results and their subsequent analyses are tantalising in that they promise to bridge the gap between the biological and physical approaches to self-organising phenomena.

When discussing the origin of the reported 1/*f* noise, we acknowledge that this noise may reflect fluctuations in membrane conductivity in case of a single pollen tube or fluctuations in external ionic fluxes for the ensemble of pollens. Because of its connections to noise analysis and ion-channel stochasticity, self-organised criticality at the level of the fractal 1/*f* scaling appears to be a fundamental property of a normal (natural conditions) living cell. As this feature can help to facilitate the recognition of altered metabolic states, which were reliably identified from the observed dynamics, the consequences of this simple fact can be far reaching. However, the most important practical conclusion arises that the *β* exponent can reflect the state of a cell or cellular ensemble and that the precise 1/*f* noise is indicative of a "healthy" cell.

A preliminary analysis of the unicellular (gametophytic) generations of two plant species and peripheral blood cells offers a unique opportunity to propose a tempting extrapolation—cell life is a (self-organised) critical dynamic process. Cell life at the 1/*f* ridge of criticality is a result of natural selection, which it appears is most efficient for biological systems due to the equal energy and charge transfer processes in all octaves of frequency *f* to maintain the non-equilibrium critical state that seems associated with the living state of matter.

## Electronic supplementary material

Below is the link to the electronic supplementary material.

Supplementary file1 (DOCX 2721 kb)
