# Supplementary material for: Extracellular ionic fluxes suggest the basis for cellular life at the 1/f ridge of extended criticality
Source: Eur Biophys J. 2020 Mar 24;49(3):239–52. doi: 10.1007/s00249-020-01430-3 (PMC7244616; doi:10.1007/s00249-020-01430-3)
Supplement: Supplementary file 1 — Supplementary file1 (DOCX 2721 kb) [file 249_2020_1430_MOESM1_ESM.docx]

Supplementary Materials for

Extracellular ionic fluxes suggest the basis for cellular life at the 1/*f* ridge of extended criticality

Mariusz Pietruszka and Monika Olszewska

## Table S1 Unpaired t-test results (conventional criteria) for the β exponent that were performed for multiple pollens of *Hyacinthus orientalis* L. G1 – group 1, G2 – group 2, SD – standard deviation, SEM – standard error of difference; n – number of experiments. “Normal” denotes the isotonic conditions at room temperature and atmospheric pressure. The row for IAA/2,4-D presents a result that was not “statistically significant” as was expected.

| ***t*-test** | ***P* value and statistical significance** | **mean of G1** – **G2**  **95% confidence interval** | **mean of G1** | **SD** | **SEM** |
| --- | --- | --- | --- | --- | --- |
|  |  |  | **mean of G2** |  |  |
| **normal/hyper**  *n* = 12 | The two-tailed *P* value 0.1205  not quite statistically significant | 0.0469  -0.0133 – 0.1071 | 0.9985 | 0.0769 | 0.0222 |
|  |  |  | 0.9517 | 0.0648 | 0.0187 |
| **normal/hypo**  *n* = 12 | The two-tailed *P* value 0.0018   very statistically significant | 0.1239  0.0515 – 0.1963 | 0.9985 | 0.0769 | 0.0222 |
|  |  |  | 0.8747 | 0.0932 | 0.0269 |
| **normal/2,4-D**  *n* = 12 | The two-tailed *P* value 0.0001  extremely statistically significant | 0.1448   0.0891 – 0.2006 | 0.9985 | 0.0769 | 0.0222 |
|  |  |  | 0.8537 | 0.0525 | 0.0152 |
| **normal/IAA**  *n* = 12 | The two-tailed *P* value 0.0001  extremely statistically significant | 0.1463  0.0843 – 0.2084 | 0.9985 | 0.0769 | 0.0222 |
|  |  |  | 0.8522 | 0.0693 | 0.0200 |
| **IAA/2,4-D**  *n* = 12 | The two-tailed *P* value 0.9524 not statistically significant | -0.0015  -0.0536 – 0.0506 | 0.8522 | 0.0693 | 0.0200 |
|  |  |  | 0.8537 | 0.0525 | 0.0152 |
| **normal/cold**  *n* = 12/*n* = 6 | The two-tailed *P* value 0.0001  extremely statistically significant | 0.1950  0.1242 – 0.2658 | 0.9985 | 0.0769 | 0.0222 |
|  |  |  | 0.8035 | 0.0356 | 0.0146 |

**Table S2** ANOVA Test. Input data on k = 6 independent treatments that were performed for multiple pollens of *Hyacinthus orientalis* L.

| **Treatment** | **normal** | **hyper** | **hypo** | **2,4-D** | **IAA** | **cold** |
| --- | --- | --- | --- | --- | --- | --- |
|  | **A** | **B** | **C** | **D** | **E** | **F** |
| **Input Data** | 1.1773 1.0736 1.0606 0.9368 0.9077 0.9130 1.0090 0.9976 0.9987 0.9559 0.9603 0.9987 | 0.7896 0.9025 0.9575 0.9631 0.9870 0.9471 1.0337 0.9297 0.9985 1.0252 0.9618 0.9238 | 0.7764 0.7567 0.9116 0.8194 0.8971 1.0239 0.9060 0.9874 0.9015 0.7336 0.9479 0.8584 | 0.8474 0.8579 0.8525 0.8354 0.8125 0.7943 0.7998 0.9813 0.8729 0.8428 0.8234 0.9176 | 0.9077 0.8496 0.8309 0.7366 0.7879 0.8012 0.8735 0.9892 0.8802 0.7757 0.9132 0.8738 | 0.8473 0.8140 0.7439 0.7972 0.7953 0.8165 |

**Table S3** ANOVA Test. Descriptive statistics that were performed for multiple pollens of *Hyacinthus orientalis* L.

| **Treatment** | **normal** | **hyper** | **hypo** | **2,4-D** | **IAA** | **cold** | **Pooled Total** |
| --- | --- | --- | --- | --- | --- | --- | --- |
|  | **A** | **B** | **C** | **D** | **E** | **F** |  |
| **observations (N)** | 12 | 12 | 12 | 12 | 12 | 6 | 66 |
| **sum** | 11.9892 | 11.4195 | 10.5199 | 10.2378 | 10.2195 | 4.8142 | 59.2001 |
| **mean** | 0.9991 | 0.9516 | 0.8767 | 0.8531 | 0.8516 | 0.8024 | 0.8970 |
| **sum of squares** | 12.0426 | 10.9129 | 9.3130 | 8.7647 | 8.7569 | 3.8686 | 53.6587 |
| **sample variance** | 0.0058 | 0.0042 | 0.0082 | 0.0028 | 0.0049 | 0.0012 | 0.0086 |
| **sample std. dev.** | 0.0764 | 0.0645 | 0.0908 | 0.0525 | 0.0699 | 0.0342 | 0.0926 |
| **std. dev. of mean** | 0.0221 | 0.0186 | 0.0262 | 0.0151 | 0.0202 | 0.0140 | 0.0114 |

**Table S4** ANOVA Test. One-way ANOVA of six independent treatments that were performed for multiple pollens of *Hyacinthus orientalis* L.

| **source** | **sum of squares SS** | **degrees of freedom** | **mean square MS** | **F statistic** | **p-value** |
| --- | --- | --- | --- | --- | --- |
| **treatment** | 0.2674 | 5 | 0.0535 | 11.0451 | 1.4461e-07 |
| **error** | 0.2905 | 60 | 0.0048 |  |  |
| **total** | 0.5579 | 65 |  |  |  |

**Table S5** ANOVA Test. Tukey HSD results. Multiple pollens of *Hyacinthus orientalis* L.

| **treatments pair** | **Tukey HSD Q statistic** | **Tukey HSD p-value** | **Tukey HSD inference** |
| --- | --- | --- | --- |
| **A vs B**  **normal/hyper** | 2.3635 | 0.5485053 | insignificant |
| **A vs C**  **normal/hypo** | 6.0957 | 0.0010053 | ** p<0.01 |
| **A vs D**  **normal/2,4-D** | 7.2661 | 0.0010053 | ** p<0.01 |
| **A vs E**  **normal/IAA** | 7.3420 | 0.0010053 | ** p<0.01 |
| **A vs F**  **normal/cold** | 7.9970 | 0.0010053 | ** p<0.01 |
| **D vs E**  **2,4-D/IAA** | 0.0759 | 0.8999947 | insignificant |

**Table S6** ANOVA Test. Scheffé results. Multiple pollens of *Hyacinthus orientalis* L.

| **treatments pair** | **Scheffé TT-statistic** | **Scheffé p-value** | **Scheffé inference** |
| --- | --- | --- | --- |
| **A vs B**  **normal/hyper** | 1.6713 | 0.7311819 | insignificant |
| **A vs C**  **normal/hypo** | 4.3103 | 0.0053753 | ** p<0.01 |
| **A vs D**  **normal/2,4-D** | 5.1379 | 0.0004390 | ** p<0.01 |
| **A vs E**  **normal/IAA** | 5.1916 | 0.0003694 | ** p<0.01 |
| **A vs F**  **normal/cold** | 5.6548 | 8.0051e-05 | ** p<0.01 |
| **D vs E**  **2,4-D/IAA** | 0.0537 | 1.0000000 | insignificant |

**Table S7** ANOVA Test that was performed for multiple pollens of *Hyacinthus orientalis* L. Bonferroni and Holm results: pairs were compared simultaneously.

| **treatments pair** | **Bonferroni and Holm TT-statistic** | **Bonferroni p-value** | **Bonferroni inference** | **Holm p-value** | **Holm inference** |
| --- | --- | --- | --- | --- | --- |
| **A vs B**  **normal/hyper** | 1.6713 | 1.4981500 | insignificant | 0.5992600 | insignificant |
| **A vs C**  **normal/hypo** | 4.3103 | 0.0009233 | ** p<0.01 | 0.0007386 | ** p<0.01 |
| **A vs D**  **normal/2,4-D** | 5.1379 | 4.7788e-05 | ** p<0.01 | 4.1417e-05 | ** p<0.01 |
| **A vs E**  **normal/IAA** | 5.1916 | 3.9185e-05 | ** p<0.01 | 3.6573e-05 | ** p<0.01 |
| **A vs F**  **normal/cold** | 5.6548 | 6.8950e-06 | ** p<0.01 | 6.8950e-06 | ** p<0.01 |
| **D vs E**  **2,4-D/IAA** | 0.0537 | 14.3604693 | insignificant | 0.9573646 | insignificant |

**Table S8** ANOVA Test. Bonferroni and Holm results: only pairs relative to A (normal) were compared simultaneously. Multiple pollens of *Hyacinthus orientalis* L.

Calculated using [https://astatsa.com/OneWay_ANOVA_with_TukeyHSD/](https://astatsa.com/OneWay_Anova_with_TukeyHSD/)

Accessed 4 October 2019.

| **treatments pair** | **Bonferroni and Holm TT-statistic** | **Bonferroni p-value** | **Bonferroni inference** | **Holm p-value** | **Holm inference** |
| --- | --- | --- | --- | --- | --- |
| **A vs B**  **normal/hyper** | 1.6713 | 0.4993833 | insignificant | 0.0998767 | insignificant |
| **A vs C**  **normal/hypo** | 4.3103 | 0.0003078 | ** p<0.01 | 0.0001231 | ** p<0.01 |
| **A vs D**  **normal/2,4-D** | 5.1379 | 1.5929e-05 | ** p<0.01 | 9.5577e-06 | ** p<0.01 |
| **A vs E**  **normal/IAA** | 5.1916 | 1.3062e-05 | ** p<0.01 | 1.0449e-05 | ** p<0.01 |
| **A vs F**  **normal/cold** | 5.6548 | 2.2983e-06 | ** p<0.01 | 2.2983e-06 | ** p<0.01 |

**Table S9** The average value (mean) of the spectral exponent (β) for different plant species (normal state). PSD ~ 1/*f* ^β^, where *f* stands for the frequency; mean β = <β> and SD – standard deviation; n – number of experiments.

| **Species** (n = 6) | **Mean β** | **SD β** | **Temperature [^○^C]** |
| --- | --- | --- | --- |
| *Hyacinthus orientalis L.* | 0.9985 | ± 0.08 | 25.12 ± 1.10 |
| *Nicotiana tabacum* L. | 0.9837 | ± 0.16 | 24.85 ± 0.98 |

**Table S10** The largest Lyapunov exponent (LLE) and EZ entropy. LLE calculated (number of iterations = 20) with the use of Lyarosenstein Matlab code [ML], while the fuzzy entropy calculated with the use of EZ entropy software [EZentropy].

| **Treatment** | | **Temperature [^○^C]** | **Spectral exponent β** | **LLE** | **Normalised**  **LLE** | **Fuzzy Entropy** |
| --- | --- | --- | --- | --- | --- | --- |
| ***Hyacinthus orientalis* L.** | |  |  |  |  |  |
| **Multi-pollens** | **Normal** | 25.3 ± 0.5 | 1.074 ± 0.024 | -15.95 | -0.9851 | 0.1743 |
|  | **Hyper** | 24.7 ± 0.5 | 0.958 ± 0.025 | -15.98 | -0.9870 | 0.2358 |
|  | **Hypo** | 24.5 ± 0.5 | 0.887 ± 0.025 | -15.99 | -0.9876 | 0.1624 |
|  | **2,4D** | 24.3 ± 0.5 | 0.853 ± 0.024 | -16.17 | -0.9987 | 0.5631 |
|  | **IAA** | 25.5 ± 0.5 | 0.850 ± 0.026 | -16.19 | -1 | 0.1274 |
|  | **Cold** | 15.8 ± 0.5 | 0.814 ± 0.024 | -16.11 | -0.9951 | 0.5923 |

[ML] [https://www.mathworks.com/matlabcentral/fileexchange/38424-largest-lyapunov-exponent-with-rosenstein-s-algorithm. Accessed 17.01.2020](https://www.mathworks.com/matlabcentral/fileexchange/38424-largest-lyapunov-exponent-with-rosenstein-s-algorithm.%20Accessed%2017.01.2020) by M. Lipowczan.

[EZentropy] Peng Li: EZ Entropy: a software application for the entropy analysis of physiological time series, Li ﻿BioMed Eng OnLine (2019) 18:30. Courtesy of the author. <https://doi.org/10.1186/s12938-019-0650-5>

**_
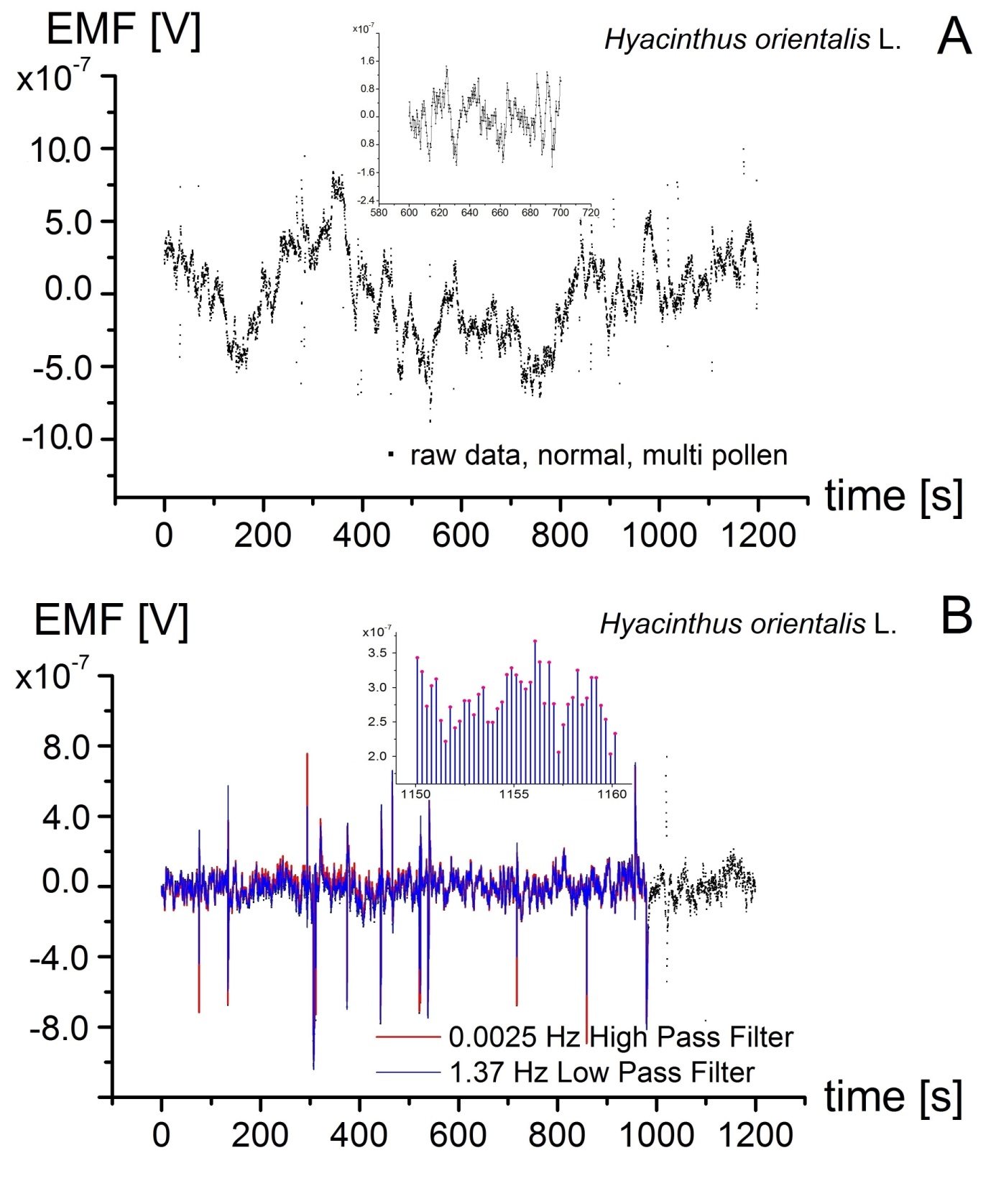
_**

**Figure S1** Representative time series of the electromotive force (EMF). **A** Raw data (5000 points) amplitude registered by the ELoPvC in the experiment on an intact *Hyacinthus orientalis* L. pollen (24.5 ± 0.5°C) at a μV level (**Inset** A one-hundred-second fragment showing dense sampling 4.1 Hz of the voltage). The linear base line was subtracted (detrended) and **B** the low-pass 1.37 Hz and high-pass 0.0025 Hz filter were applied. **Inset** Stable fluctuations in the 10 s envelope of oscillations.

**
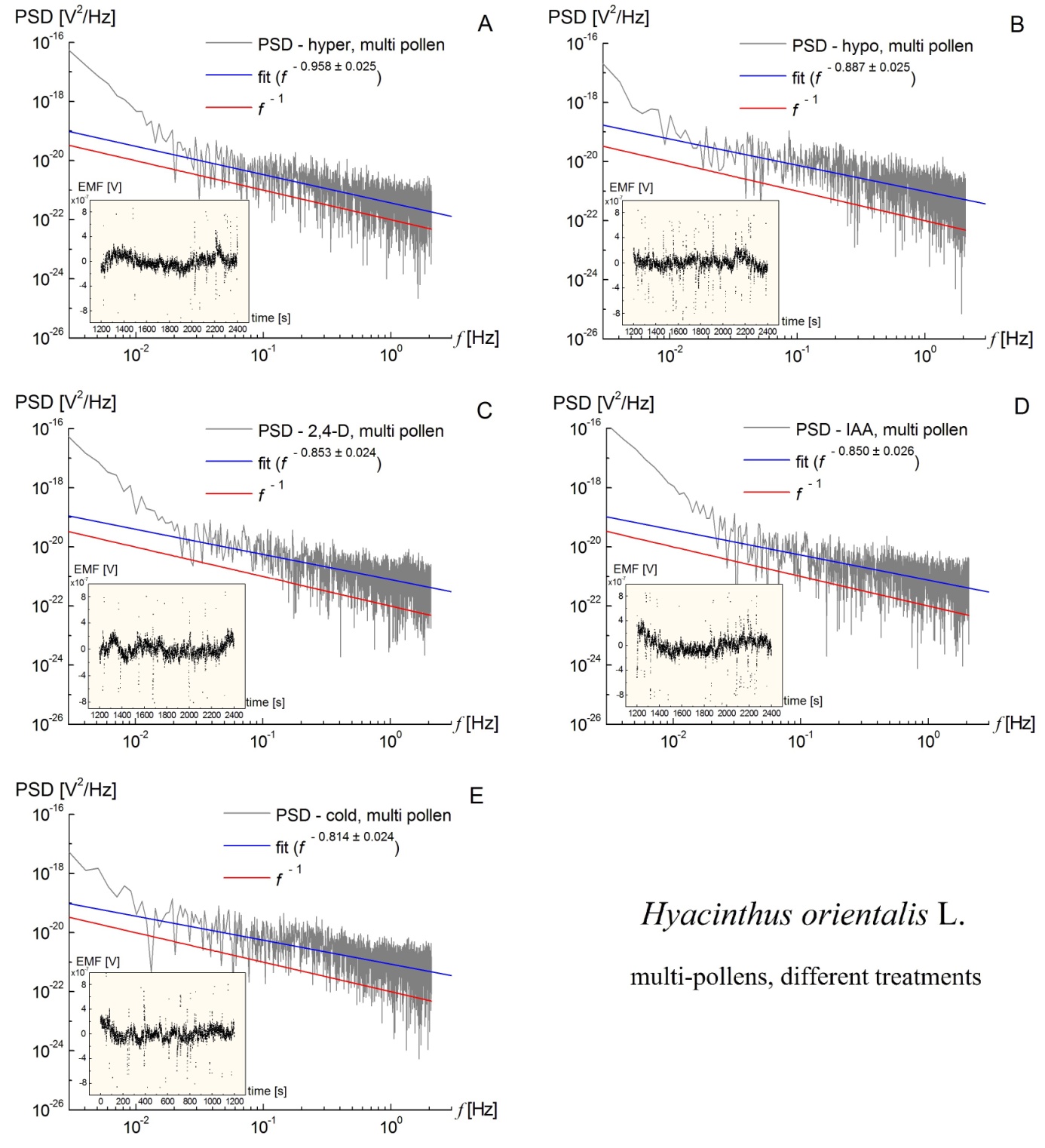
**

**Figure S2** Representative power spectral density as a function of the frequency that was calculated for the EMF that was induced by the ionic fluxes of a group of pollens of *Hyacinthus orientalis* L. **A** Hypertonic treatment at ~24.7 ± 0.5°C; the actual fit β = 0.958 ± 0.025 is indicated by the blue line. **B** Hypotonic treatment at ~24.5 ± 0.5°C; β = 0.887 ± 0.025. **C** 2,4-D ~24.3 ± 0.5°C; β = 0.853 ± 0.024. **D** IAA ~25.5 ± 0.5°C; β = 0.850 ± 0.026. **E** Cold ~15.8 ± 0.5°C; β = 0.814 ± 0.024. The insets represent the signal that was obtained. The precise 1/*f* behaviour is indicated by the straight red line for comparison (the irrelevant intercept was neglected).

**
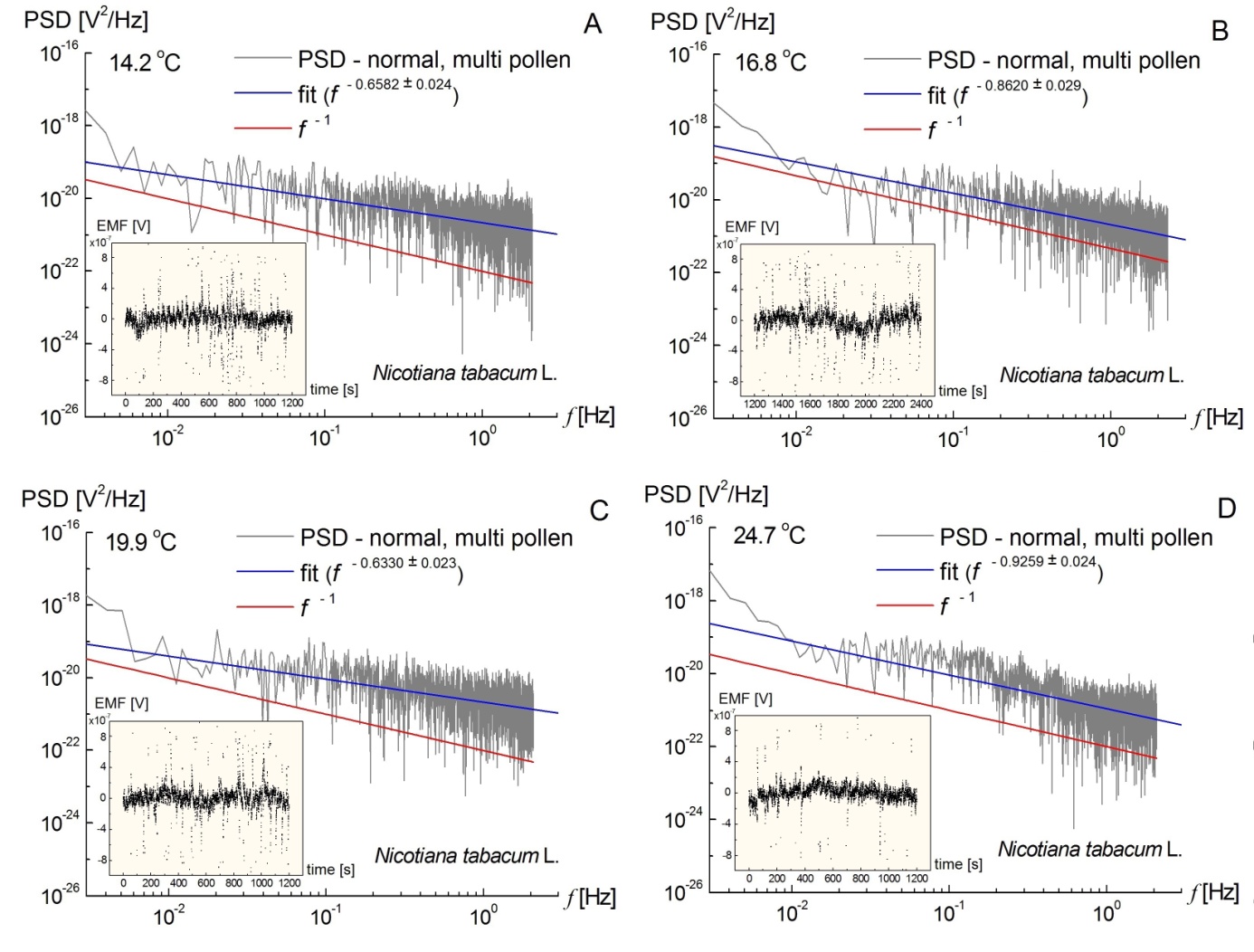
**

**Figure S3** Representative flicker noise spectrum as a function of the frequency that was calculated for the EMF that was induced by the extracellular ionic fluxes of a group of pollens of *Nicotiana tabacum* L. parameterised by temperature: **A** 12.2 ± 0.5°C, **B** 16.8 ± 0.5°C, **C** 19.9 ± 0.5°C and **D** 24.7 ± 0.5°C. The actual fits are indicated by the blue line.

**T-test for the normal state mean of the β exponent for *Hyacinthus orientalis* L.**

We conducted a t-test for one population mean with an unknown population standard deviation, which is why the sample standard deviation (s) was used instead. The provided sample mean was <β> = 0.9985, the sample standard deviation was s = 0.08 and the sample size was n = 12.

The following null and alternative hypotheses needed to be tested:

H_0_: <β>_pop_ = 1

H_a_: <β>_pop_ ≠ 1

This corresponded to a two-tailed test for which a t-test for one mean with unknown population standard deviation was used.

Based on the information provided, the significance level was α = 0.001 and the critical value for a two-tailed test was t_c_ = 4.437. The rejection region for this two-tailed test was *R* = {*t*: ∣*t*∣ > 4.437}.

The t-statistic was computed as follows:

$$t=\frac{<\beta>-\mu_{0}}{s\sqrt{n}}=-0.065$$

Since it was observed that ∣*t*∣ = 0.065 ≤ t_c_ = 4.437, it was then concluded that *the null hypothesis was not rejected.* Therefore, there was not enough evidence to claim that the population mean <β>_pop_ was different than 1 at the 0.001 significance level. The 99.9% confidence interval was 0.896 < *<*β>_pop_ < 1.101.


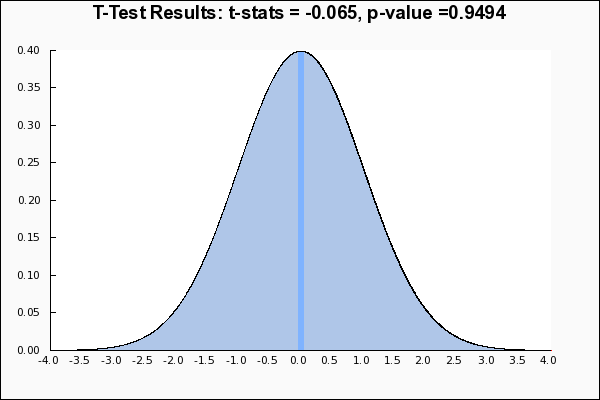


**Figure S4** T-test results for the mean of the β exponent of *Hyacinthus orientalis* L. (normal state). Calculated using <https://mathcracker.com/t-test-for-one-mean.php#results>

Accessed 10 October 2019.

**T-test for the normal state mean of the β exponent for *Nicotiana tabacum* L.**

We conducted a t-test for one population mean with an unknown population standard deviation, which is why the sample standard deviation (s) was used instead. The provided sample mean was <β> = 0.9837, the sample standard deviation was s = 0.16 and the sample size was n = 12.

The following null and alternative hypotheses needed to be tested:

H_0_: <β>_pop_ = 1

H_a_: <β>_pop_ ≠ 1

This corresponded to a two-tailed test for which a t-test for one mean with an unknown population standard deviation was used.

Based on the information provided, the significance level was α = 0.001 and the critical value for a two-tailed test was t_c_ = 4.437. The rejection region for this two-tailed test was *R* = {*t*: ∣*t*∣ > 4.437}.

The t-statistic was computed as follows:

$$t=\frac{<\beta>-\mu_{0}}{s\sqrt{n}}=-0.353$$

Since it was observed that ∣*t*∣ = 0.353 ≤ t_c_ = 4.437, it was then concluded that *the null hypothesis was not rejected.* Therefore, there was not enough evidence to claim that the population mean <β>_pop_ was different than 1 at the 0.001 significance level. The 99.9% confidence interval was 0.779 < *<*β>_pop_ < 1.189.


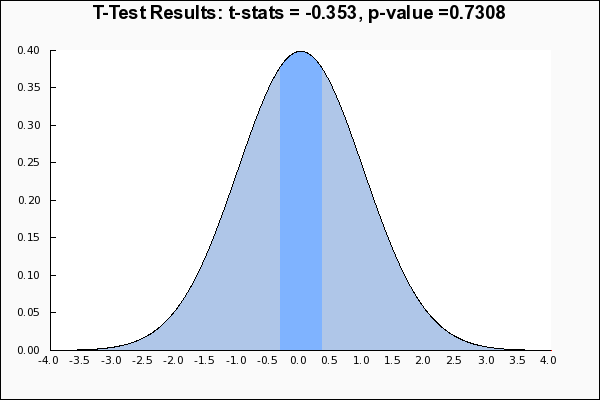


**Figure S5** T-test results for the mean of the β exponent of *Nicotiana tabacum L*. (normal state). Calculated using <https://mathcracker.com/t-test-for-one-mean.php#results>

Accessed 10 October 2019.

**
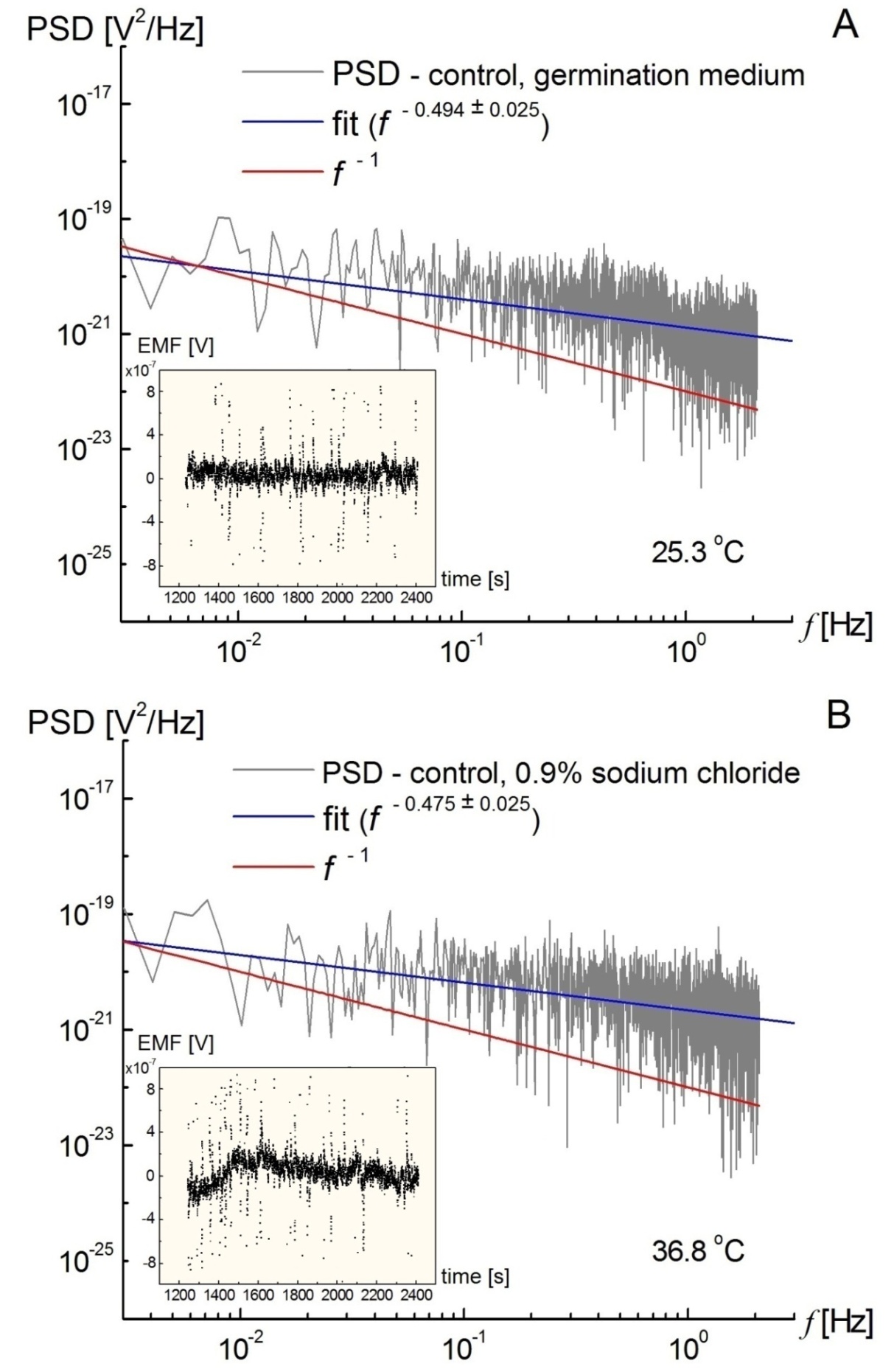
**

**Figure S6** Power spectral density as a function of the frequency at control conditions. **A** Germination medium at 25.3 ± 0.5°C: *f* ^-0.494 ± 0.025^. **B** 0.9% sodium chloride at 36.8 ± 0.5°C:

*f* ^-0.475 ± 0.024^. The insets represent the signal that was obtained.

**
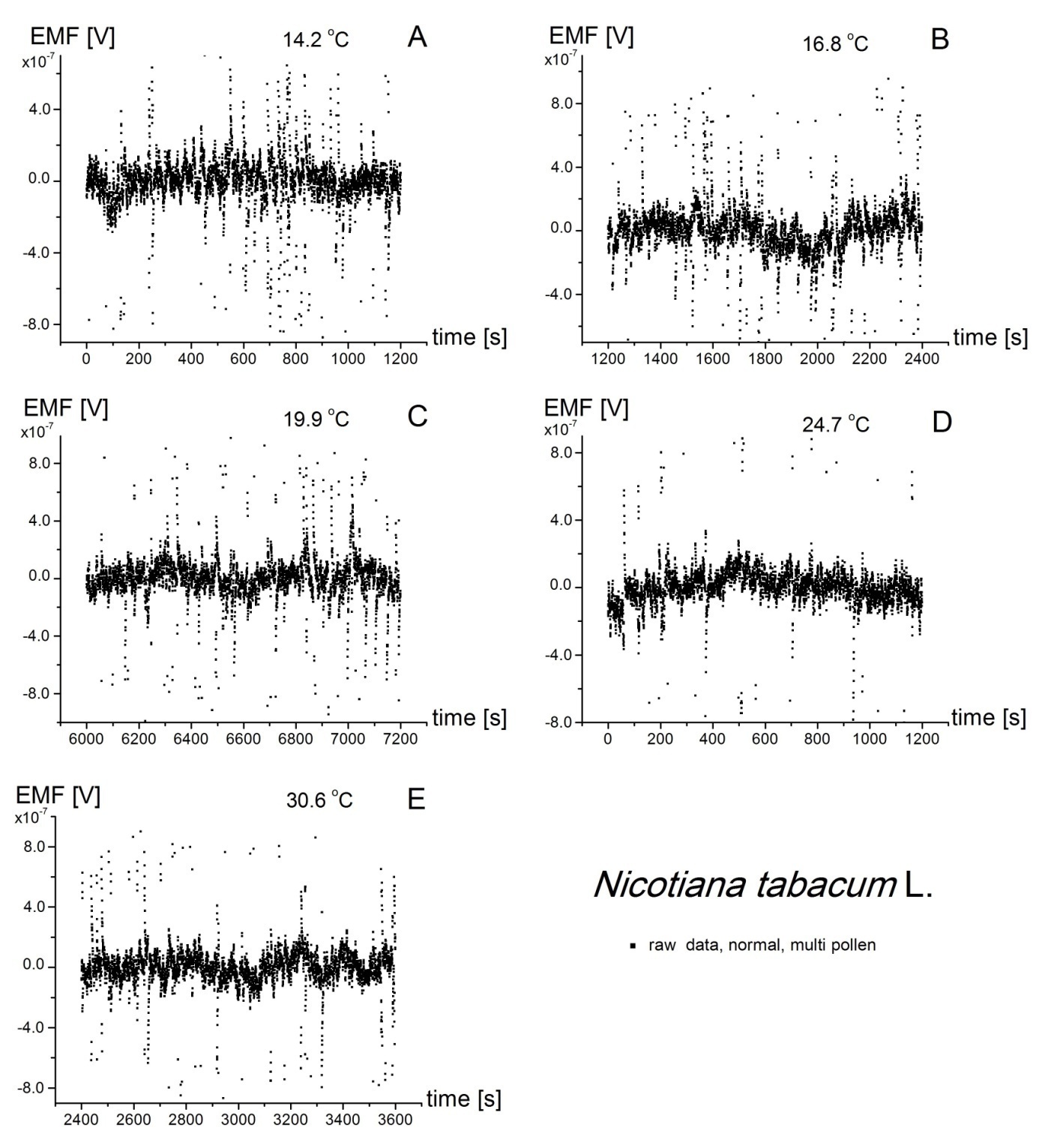
**

**Figure S7** Representative time series of the electromotive force (EMF) parameterised by temperature. Raw data (total 5000 points for each 20 min. phase, sampling at 4.1 Hz). The amplitude was registered by an ELoPvC in the experiment on intact *Nicotiana tabacum* L. pollen tubes. The linear base line was subtracted (detrended).


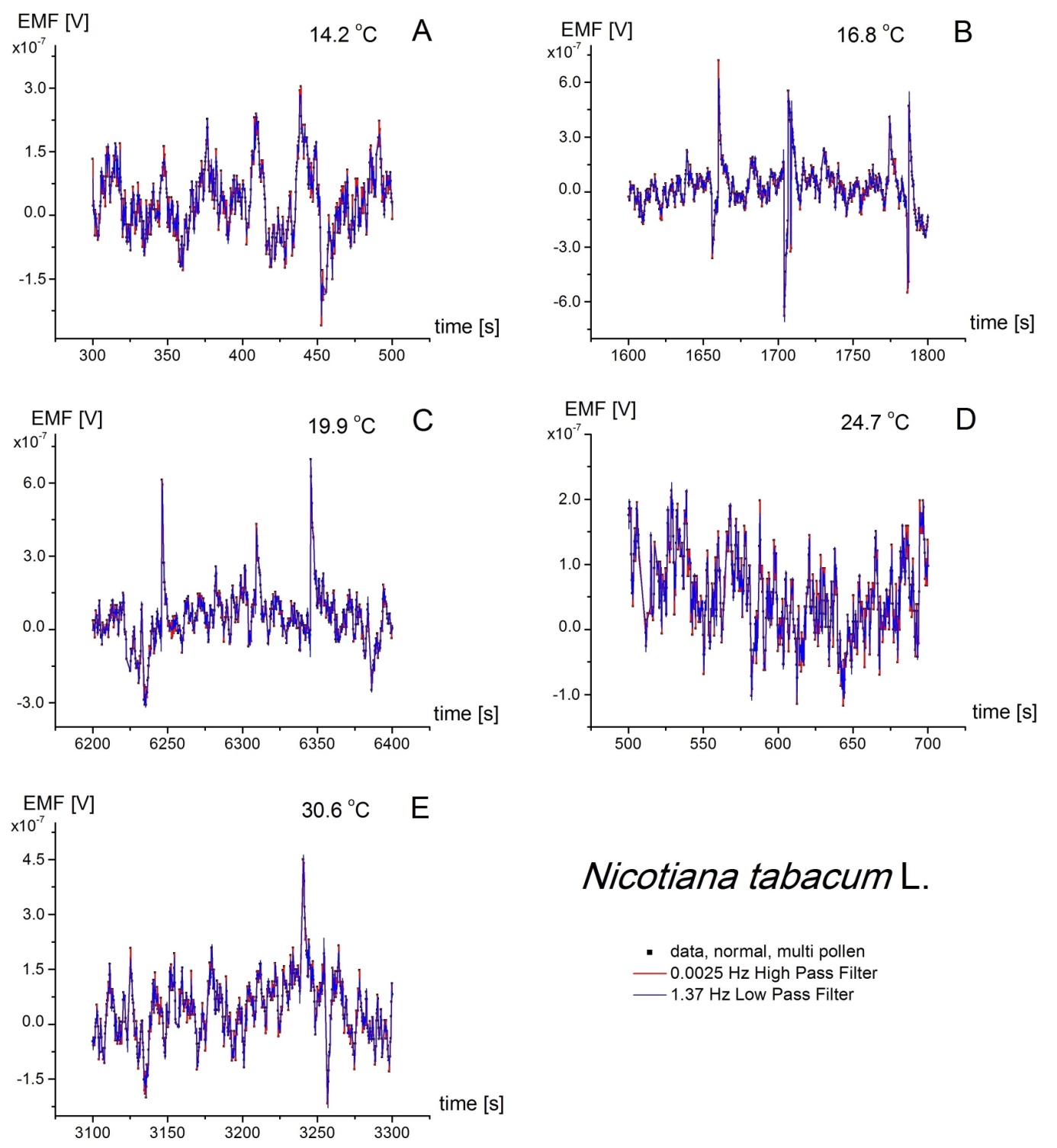


**Figure S8** Representative fragments (zoomed in 200 s) time series of the electromotive force (EMF) parameterised by temperature. The raw data that were sampled at 4.1 Hz were registered by an ELoPvC in the experiment on intact *Nicotiana tabacum* L. pollen tubes on μV level. The linear base line was subtracted (detrended) and the low-pass 1.37 Hz and high-pass filter 0.0025 Hz were applied. See also Figure S7 for the whole range.


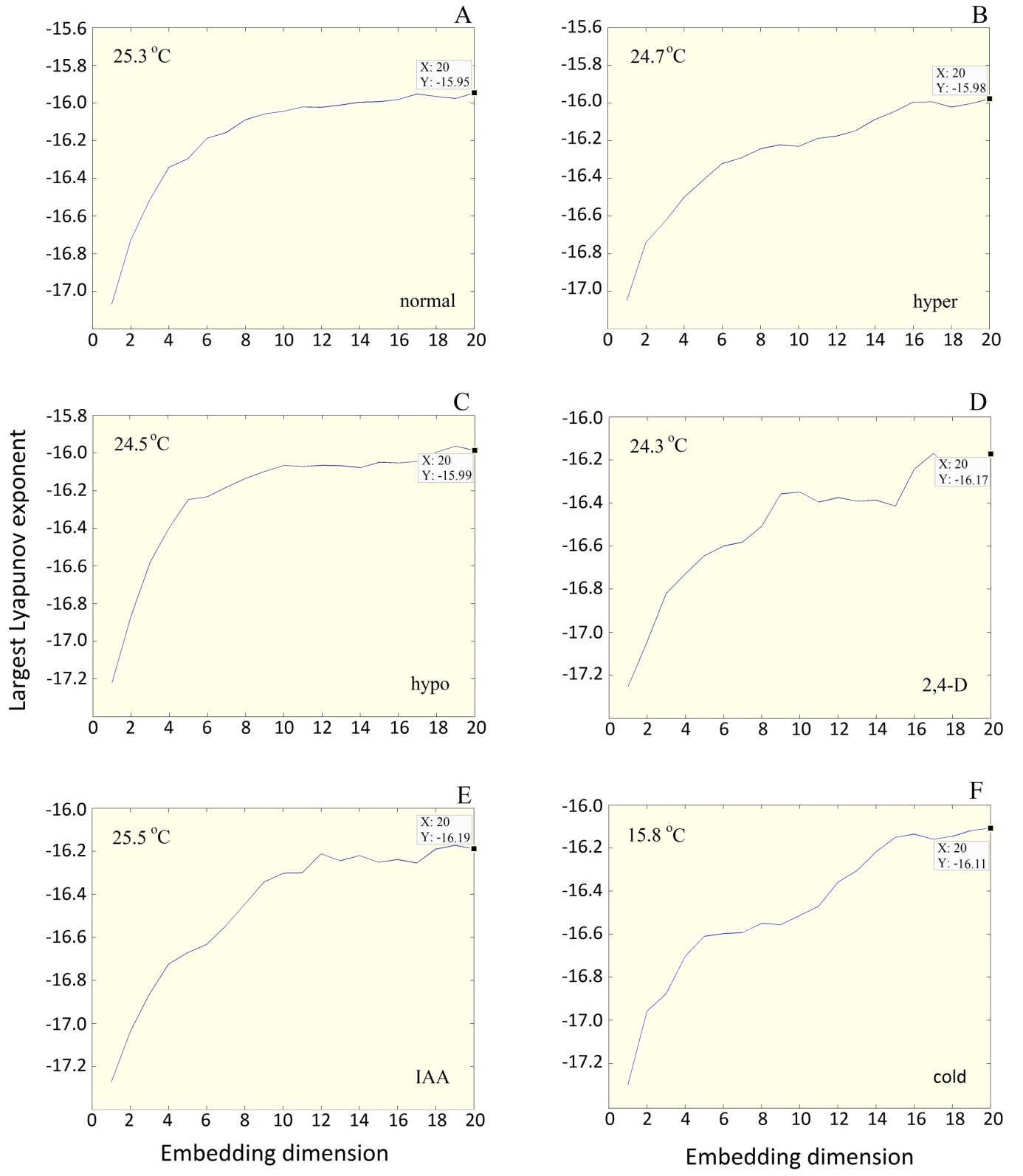


**Figure S9** Largest Lyapunov exponent as a function of embedding dimension for multi-pollens of *Hyacinthus orientalis* L. for different treatments.

**
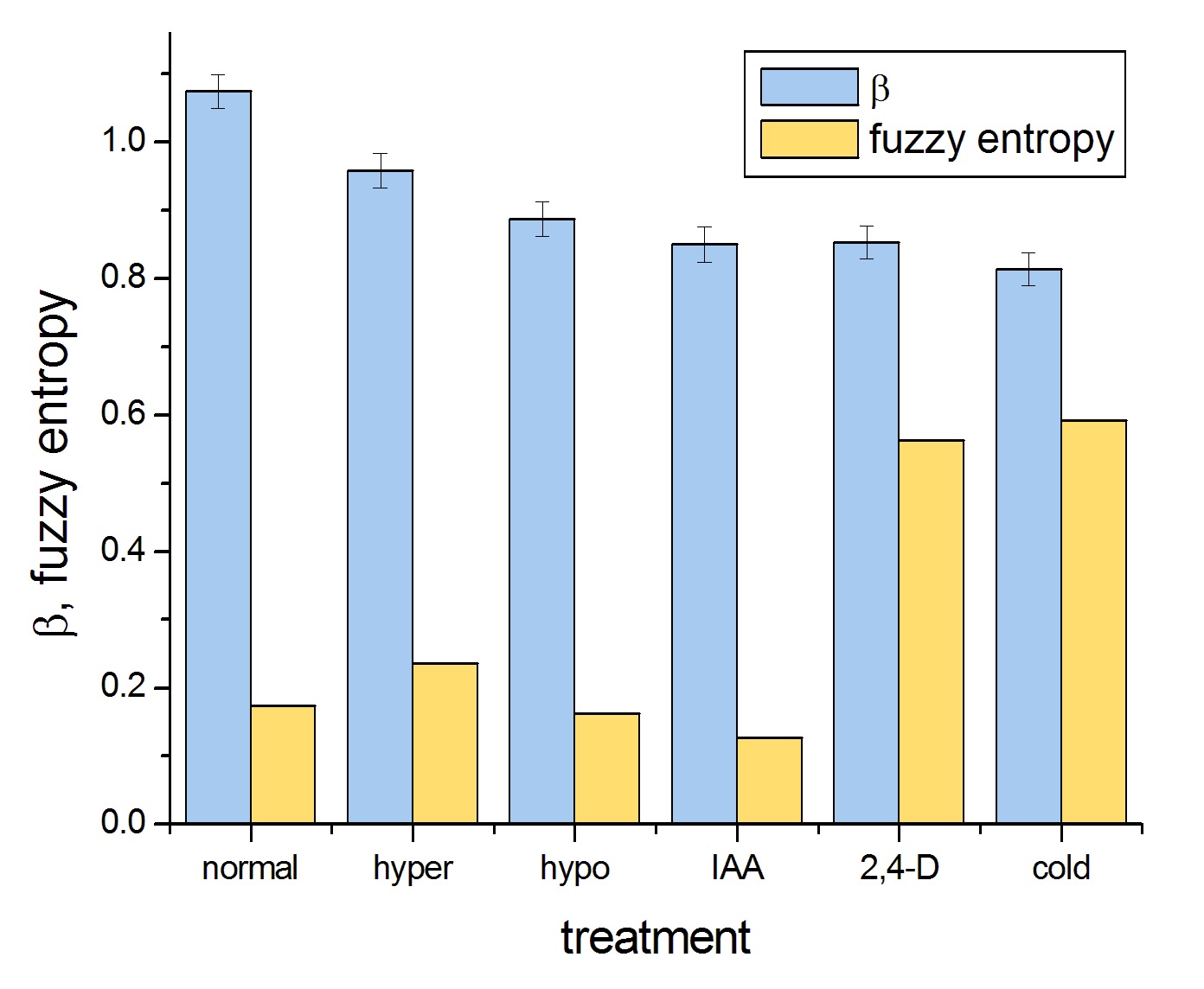
**

**Figure S10** EZ entropy and the spectral exponent β calculated for different treatments of *Hyacinthus orientalis* L. multi-pollens (Table S10).


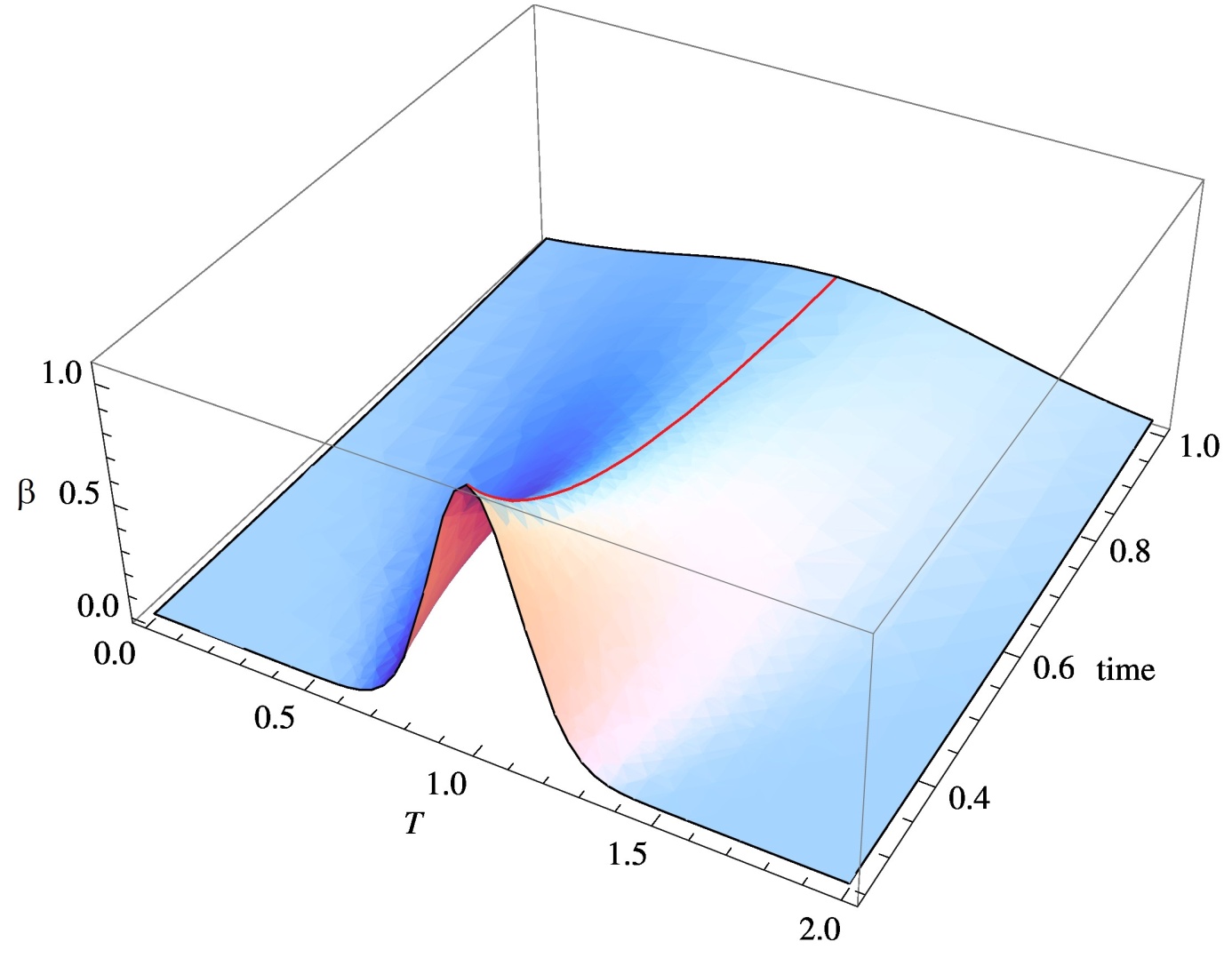


**Figure S11** Critical exponent β as a function of temperature (*T*) and time (both in a.u.). An illustration for the ridge (red line) of extended criticality.
